# The oncogenic kinase NEK2 regulates an RBFOX2-dependent pro-mesenchymal splicing program in triple-negative breast cancer cells

**DOI:** 10.1186/s13046-021-02210-3

**Published:** 2021-12-20

**Authors:** Chiara Naro, Monica De Musso, Francesca Delle Monache, Valentina Panzeri, Pierre de la Grange, Claudio Sette

**Affiliations:** 1 Department of Neuroscience, Section of Human Anatomy, Catholic University of the Sacred Heart, 00168 Rome, Italy; 2 Fondazione Policlinico Universitario A. Gemelli, IRCCS, Rome, Italy; 3 Genosplice, Paris, France; 4 Fondazione Santa Lucia, IRCCS, Rome, Italy

**Keywords:** Triple-negative breast cancer, Alternative splicing, NEK2, Breast-cancer prognosis, EMT

## Abstract

**Background:**

Triple-negative breast cancer (TNBC) is the most heterogeneous and malignant subtype of breast cancer (BC). TNBC is defined by the absence of expression of estrogen, progesterone and HER2 receptors and lacks efficacious targeted therapies. NEK2 is an oncogenic kinase that is significantly upregulated in TNBC, thereby representing a promising therapeutic target. NEK2 localizes in the nucleus and promotes oncogenic splice variants in different cancer cells. Notably, alternative splicing (AS) dysregulation has recently emerged as a featuring trait of TNBC that contributes to its aggressive phenotype.

**Methods:**

To investigate whether NEK2 modulates TNBC transcriptome we performed RNA-sequencing analyses in a representative TNBC cell line (MDA-MB-231) and results were validated in multiple TNBC cell lines. Bioinformatics and functional analyses were carried out to elucidate the mechanism of splicing regulation by NEK2. Data from The Cancer Genome Atlas were mined to evaluate the potential of NEK2-sensitive exons as markers to identify the TNBC subtype and to assess their prognostic value.

**Results:**

Transcriptome analysis revealed a widespread impact of NEK2 on the transcriptome of TNBC cells, with 1830 AS events that are susceptible to its expression. NEK2 regulates the inclusion of cassette exons in splice variants that discriminate TNBC from other BC and that correlate with poor prognosis, suggesting that this kinase contributes to the TNBC-specific splicing program. NEK2 elicits its effects by modulating the expression of the splicing factor RBFOX2, a well-known regulator of epithelial to mesenchymal transition (EMT). Accordingly, NEK2 splicing-regulated genes are enriched in functional terms related to cell adhesion and contractile cytoskeleton and NEK2 depletion in mesenchymal TNBC cells induces phenotypic and molecular traits typical of epithelial cells. Remarkably, depletion of select NEK2-sensitive splice-variants that are prognostic in TNBC patients is sufficient to interfere with TNBC cell morphology and motility, suggesting that NEK2 orchestrates a pro-mesenchymal splicing program that modulates migratory and invasive properties of TNBC cells.

**Conclusions:**

Our study uncovers an extensive splicing program modulated by NEK2 involving splice variants that confer an invasive phenotype to TNBCs and that might represent, together with NEK2 itself, valuable therapeutic targets for this disease.

**Supplementary Information:**

The online version contains supplementary material available at 10.1186/s13046-021-02210-3.

## Background

Breast cancer (BC) is the most common malignancy and the second cause of cancer-related death among women worldwide [1]. BC is a heterogeneous disease that comprises multiple subtypes, showing different histopathological traits, molecular alterations and clinical course [1, 2]. Identification of estrogen (ER), progesterone (PR) and human epidermal growth factor receptor 2 (HER2) receptors as molecular markers and oncogenic drivers for the disease has allowed more accurate stratification of tumours and development of efficacious targeted therapies, leading to significant improvements in the management and outcome of BC patients [1, 2]. Nevertheless, ~ 15% of BCs, called triple-negative breast cancers (TNBCs), lack the expression of these receptors and cannot benefit from endocrine and anti-HER2 therapies. Standard chemotherapy remains the only established therapeutic option for TNBC patients [3]. However, despite the initial response to chemotherapy, most TNBC patients display poor outcome compared to other BC subtypes, primarily due to their higher relapse rate and earlier onset of metastasis [3]. Thus, identification of molecular determinants of the aggressive phenotype of TNBCs represents a clinical priority for the development of improved prognostic tools and of novel therapeutic strategies.

In the last decade, employment of transcriptome profiling by RNA-sequencing (RNA-seq) analyses has represented a striking breakthrough in cancer research [4]. Comprehensive expression profiling of cancer cells has highlighted hallmarks that clearly differentiate tumours previously thought to be similar [4]. In particular, a recent survey of datasets obtained from more than 8000 patients affected by different cancer types revealed that tumours display considerably wider transcriptome diversity than corresponding normal tissues [5]. Such increased complexity is mainly caused by widespread dysregulation of alternative splicing (AS), which often yields oncogenic variants that promote tumorigenesis and resistance to treatments [6–8]. On the other hand, aberrant splicing can also represent a vulnerability of the tumors that can be exploited therapeutically. For instance, splicing defects can create tumour-specific neo-junctions that encode variant-specific peptides or neoepitopes, which may be used to develop anti-cancer immunotherapy [9]. BC also displays a specific splicing signature with respect to normal mammary tissue [10]. Moreover, integration of transcriptomic and clinical data from patients has revealed that splice-variants represent powerful and robust markers for both prognosis and precise discrimination of TNBCs from “other BC” subtypes [11–13]. These studies clearly highlighted splicing dysregulation as a common trait in TNBC, which likely contributes to its aggressive phenotype. Thus, characterization of the splicing regulators involved in the aberrant regulation of this process might offer new therapeutic perspective to understand the highly malignant nature of this cancer.

AS is a combinatorial post-transcriptional process mediated by the spliceosome, the molecular machinery that excises intron and ligates exons in the pre-mRNA, and multiple auxiliary factors [7, 8]. AS expands the coding potential of the genome by allowing most eukaryotic genes to produce multiple transcripts that encode proteins with potentially different functions. Furthermore, the flexible nature of AS represents a key tool for the control of gene expression, which finely controls several cellular functions and cell-differentiation processes in response to external and internal cues [14]. On the other hand, defective AS regulation can promote neoplastic transformation through generation of aberrantly spliced variants [7, 8]. Deregulated expression and activity of splicing factors that cooperate with the spliceosome are often the cause of AS dysregulation in cancer [7, 8]. For instance, overexpression of the serine and arginine rich splicing factor 1 (SRSF1) is sufficient to drive oncogenic transformation of epithelial mammary cells by promoting splice-variants with anti-apoptotic functions [15]. Another major class of regulatory proteins of pre-mRNA processing is represented by splicing factor kinases, whose altered expression was largely shown to concur to the aberrant AS patterns of cancer cells [16]. In particular, both canonical and non-canonical splicing factors kinases, such as SRSF protein kinase 1 (SRPK1) or cyclin dependent kinase 12 (CDK12), were shown to be aberrantly expressed in BC cells and to promote tumorigenesis through AS regulation [17, 18].

An oncogenic kinase that is frequently upregulated in cancer, including BC, is the NIMA related kinase 2 (NEK2) [19–21]. NEK2 is a mitotic kinase, whose high levels of expression have been associated with poor prognosis in BC patients [20, 22]. The oncogenic activity of NEK2 in BC has been primarily linked to regulation of centrosome splitting during mitosis [19, 20, 23], which was shown to underlie the sensitizing effect of NEK2-targeting approaches to chemotherapeutic and microtubule-poisoning agents [23, 24]. However, more recent evidence indicates that this kinase accumulates in the nucleus of cancer cells [22, 25], interacts with splicing factors and promotes pro-tumoral splice-variants [26, 27], but the possible function of NEK2 in the nucleus of BC cells is currently unexplored.

In this study, we found that NEK2 is enriched in the nucleus of TNBC cells with respect to other BC types. Moreover, transcriptome analysis of TNBC cells transiently silenced for NEK2 expression uncovered a widespread impact of this kinase on AS regulation in TNBC, with more than thousand NEK2-regulated exons. Cassette exons that are sensitive to NEK2 depletion significantly overlap with the splicing signature discriminating TNBC from other BC, as well as with the splicing program activated in BC cells during epithelial to mesenchymal transition (EMT). Furthermore, our results indicate that NEK2 modulates the expression of the splicing factor RNA binding fox-1 homolog 2 (RBFOX2), a key regulator of the EMT process. Notably, depletion of select NEK2-sensitive splice-variants interferes with the mesenchymal phenotype of TNBC cells and reduces their motility, indicating that NEK2 orchestrates a pro-mesenchymal splicing program that modulates migratory and invasive properties of TNBC cells. Thus, our study uncovers a role for NEK2 in the regulation of the TNBC transcriptome and hints at the involvement of this activity in the aggressive and metastatic behaviour of TNBC.

## Methods

### Cell culture, treatment and transfection

ZR-75-1, MCF-7, SK-BR-3, MDA-MB-231, MDA-MB-468, MDA-MB-436, HCC1806, HCC1937 cells were grown in RPMI 1640 (Lonza), SUM159 were grown in DMEM/F12(Sigma Aldrich), all supplemented with 10% FBS, gentamycin, penicillin and streptomycin. For RNA interference, cells were transfected with siRNAs (Sigma-Aldrich) using Lipofectamine RNAiMAX (Invitrogen) according to manufacturer’s instructions and harvested 48 h later for protein and RNA analyses. For RNA stability assay, 48 h after silencing, cells were treated with either Actinomycin D (3 μg/ml) or DMSO and collected for RNA analyses at indicated time points. Sequences of siRNAs are listed in Additional File 1: Supplemental Table 1.

### Cell proliferation assay

Transiently silenced MCF-7 and MDA-MB-231 cells were seeded in a 96 well plate and imaged at 10X magnification in an IncuCyte SX5 Live-content imaging system (Essen Bioscience) at 37 °C with 5% CO2. Images were acquired every 24 h for 4 days (4 images/well). Data were analysed using IncuCyte Cell-by-Cell analysis software to detect and quantify live cell (phase-contrast).

### RNA extraction, library preparation and RNA-Seq data analysis

For RNA-seq analysis, MDA-MB-231 transiently transfected with control (si-CTRL) or NEK2-targeting (si-NEK2) pool of siRNAs were harvested 48 h after transfection in triplicate and total RNA was extracted and DNAse treated using the RNEasy mini kit (Qiagen) according to manufacturer’s instruction. PolyA plus RNA-seq libraries were constructed according to Illumina’s protocol and sequenced using a 100 bp single-end format on an Illumina HiSeq 2000. RNA-Seq data analysis was performed by GenoSplice technology (www.genosplice.com), as previously described [28], using Human FAST DB v2016_1 annotations. Results were considered statistically significant for *p*-values≤0.05 and fold-changes≥1.5.

### Bioinformatic analysis

Gene expression and AS analysis of transcriptomic data of BC patients from the “The Cancer Genome Atlas (TCGA)” database [29] and of SRP042620 RNA-seq dataset [30] were performed using the visual interface of the Psichomics R package [31]. Transcriptomic data from the METABRIC project [32] were analysed using the cBioPortal resource [33, 34]. Other indicated microarray data of primary BC specimen were analysed using the “R2: Genomics Analysis and Visualization Platform (http://r2.amc.nl)”. Expression data of the Cancer Cell Line Encyclopedia (CCLE) project [35] were downloaded from https://depmap.org/portal/. For analysis of cis-regulatory sequence and elements of NEK2-regulated cassette exons, genes expressed in MDA-MB-231 according to our RNA-seq data, but not regulated neither at the gene or exon/pattern level were considered as reference genes. Eleven thousand fifty-one single cassette exons from these genes were defined as “reference cassette exons”; 22,480 exons from these genes that are not first, not last and included in 100% of its gene transcripts according to FAST DB were defined as “reference constitutive exons”. Analysis of 3′ and 5′ splice-sites strengths and GC context of regulated exon cassette was performed as previously described [28]. Polypyrimidine tract length/score and distance of branchpoint from acceptor splice-site were evaluated using SVM-BPfinder [36]. Search for enriched motif within regulated cassette exons and their comparison with the compendium of RNA-binding motif from [37] were performed using DREME and Tomtom Motif comparison tools from the MEME Suite Collection [38, 39]. Analysis for enriched GO functional clusters among AS regulated genes was performed as previously described [28]. Clusters were considered as enriched if fold enrichment ≥2.0 and *p*-value ≤0.05.

### Extraction of RNA, RT-PCR and real-time PCR analysis

RNA-extraction, RT-PCR and real-time PCR (qRT-PCR) analysis were performed as described [26, 28]. Densitometric analysis of agarose gels were performed using Image Lab 3.0 Software (Biorad). All primers used are listed in Additional File 1: Supplemental Table 2.

### Protein extracts and Western blot analysis

Total cell extracts were obtained by lysis in RIPA buffer as described [28]. Cellular fractionation was performed as described in [40]. Western Blot were performed as described [26, 28] using following primary antibodies: mouse anti-hnRNP F/H (dilution 1:1000, Abcam), anti-VIMENTIN (dilution 1:2000, Sigma Aldrich), anti-TUBULIN (dilution 1:1000, Sigma Aldrich), anti-NEK2 (dilution 1:500, Santa Cruz), anti-NF-YA, anti-HSP90a/b, anti-GAPDH, anti-SRSF1 (dilution 1:1000, Santa Cruz); goat anti-MATRIN3 (1:500, Santa Cruz); rabbit anti-H3 (dilution 1:1000, Abcam) and anti-E-CADHERIN (1:1000, Cell Signalling). Densitometric analysis were performed using Alliance system software (UVITEC, Cambridge).

### Immunofluorescence analysis

MCF-7 and MDA-MB-231 cells were fixed and permeabilized as described [25]. Following incubation for 1 h at room temperature in blocking buffer (PBS, 5% BSA, 3% horse serum), cells were incubated over-night with anti-NEK2 antibody (Santa Cruz), diluted 1:500 in the same blocking buffer, and then 1 h at 37 °C with anti-mouse Alexa Fluor 488 (Thermo Fisher Scientific), diluted 1:400 in PBS, 1% BSA. Images were taken using a Nikon Eclipse Ti2 confocal microscope and Z stack images were processed by NIS Elements AR 5.30 software using the same acquisition settings. For cytoskeleton labeling, MDA-MB-231 control or NEK2 silenced cells, fixed and permeabilized as described above, were incubated 1 h at room temperature with FITC-conjugated phalloidin (1:100, Sigma Aldrich). Nuclei were counterstained with DAPI or Hoechst 33342. Images were taken using an Axio Observer.Z1/7 inverted microscope (Carl Zeiss) equipped with a LD Plan-Neofluar 20X 0.4 objective lens. Images were elaborated with Photoshop (Adobe) for composing panels.

### Wound healing assay

MDA-MB-231 cells were plated at 100% of confluence and a wound was created by scratching with a sterile pipette tip. Following two washes with PBS and addition of 1%-FBS supplemented medium, the plate was photographed immediately and 8 h after scratching. Area quantification of the scratch was performed with ImageJ software using the MRI Wound Healing tool.

### Cell invasion assay

MDA-MB-231 cells were seeded into the IncuCyte Clearview 96-well inserts (Sartorius; 1000 cells/well). Inserts membrane had been pre-coated on both sides with 50 μg/ml Matrigel (Corning), diluted in RPMI 1640. Lower chambers were filled with 200 μl of either chemotaxis assay medium (RPMI 1640, supplemented with 10% FBS) or negative control medium (RPMI 1640, without FBS). Images were acquired with IncuCyte SX5 Live-content imaging system every hour for 24 h at 10X magnification. Migrated cells were quantified using the IncuCyte Chemiotaxis migration software (phase-contrast; top/bottom), starting 2 h after initial seeding to allow settlement of cells.

### Quantification and statistical analysis

Statistical analyses for differential gene expression, splicing changes, comparison of different datasets, analysis of motifs and cis-acting sequence features were performed in R using the statistical tests described in the figure legends. Number of genes and sequences analysed is detailed by the “n” in each figure legend. Statistical analyses for qRT-PCR and densitometric analysis of PCR and Western blot analysis were performed in GraphPad Prism according to the statistical tests described in the figure legends. Number of replicates independently analysed is indicated by the “n” in each figure legend. Results were considered statistically significant if *p*-value ≤0.05.

## Results

### NEK2 is highly expressed in TNBC patients and cell lines

**Fig. 1:**
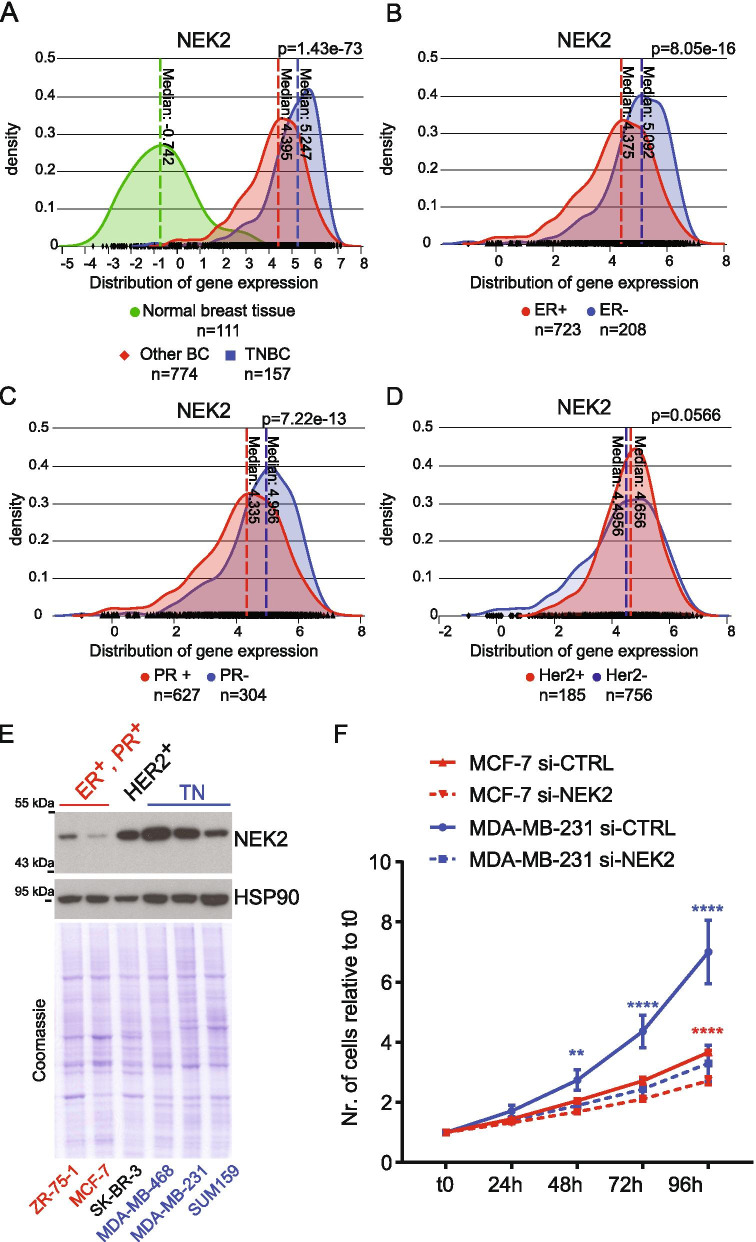
NEK2 is highly expressed in TNBC. **A-D** Analysis of TGCA data for NEK2 expression using the visual interface of the psichomics R package. **A** Density plots showing distribution of NEK2 gene expression in normal breast tissues, triple-negative (TNBC) and other breast cancer subtypes (Other BC) (indicated *p*-value estimated by Kruskal-Wallis rank sum test). **B-D** Density plots showing distribution of NEK2 gene expression in hormone positive (ER^+^, PR^+^) or negative (ER^−^, PR^−^) and HER2 amplified (HER2^+^) or HER2 negative (HER2^-^) breast cancers (indicated *p*-value estimated by Wilcoxon rank sum test with continuity correction). **E** Representative Western blot analysis of NEK2 expression in indicated hormone-positive (ER^+^PR^+^), HER2-positive (HER2^+^) or triple-negative (TN) breast cancer cell lines. HSP90 was evaluated as loading control. **F** Line graph showing growth rate of MCF-7 and MDA-MB-231 cells transfected with either control (si-CTRL) or NEK2 targeting si-RNAs (si-NEK2). Number of cells at indicated time points was evaluated by IncuCyte® SX5 Live-Cell Analysis Imaging System and normalized to t0 (mean ± SD, *n* = 4, **p ≤ 0.01, ****p ≤ 0.0001, two-way Anova)

NEK2 is frequently up-regulated in BC and promotes oncogenic features associated with poor prognosis [19, 20, 23]. To investigate whether high expression of NEK2 was particularly associated with a BC subtype, we queried data from primary tumors and normal tissues available in The Cancer Genome Atlas (TCGA). By using the psichomics R package [31], we observed that NEK2 is significantly more expressed in TNBC compared to both normal breast tissue and all other BC subtypes (Fig. 1A, Additional File 2: Supplemental Fig. 1A,B). Upregulation of NEK2 expression in TNBC compared to normal mammary tissue and other BC subtypes was also confirmed by analysis of data from five additional cohorts of BC patients (Additional File 2: Supplemental Fig. 1C-G) [32]. In particular, stratification of BC patients for receptors status revealed that NEK2 is expressed at higher levels in ER- and PR-negative tumours (Fig. 1B,C), while no significant changes were observed when patients were stratified for HER2 amplification (Fig. 1D). This pattern of differential NEK2 expression in BC subtypes was largely confirmed by analysis of data from the METABRIC project [32], even though a moderate but significant upregulation of NEK2 in HER2-positive tumors was observed in this cohort (Additional File 2: Supplemental Fig. 1H-L). In line with these observations, analysis of transcriptomic data from the Cancer Cell Line Encyclopedia (CCLE) project [35] revealed higher NEK2 expression in ER^−^/HER2^−^ cell lines compared to those representative of other BC subtypes (Additional File 2: Supplemental Fig. 1 M). Computational analyses were validated by both qRT-PCR and Western blot analysis, which confirmed higher levels of NEK2 transcript and protein in representative TNBC cell lines with respect to other BC subtypes (Fig. 1E; Additional File 2: Supplemental Fig. 1 N). Moreover, we found that while depletion of NEK2 reduced the growth rate of both TNBC (MDA-MB-231) and ER^+^/PR^+^ BC cells, its impact was much stronger in TNBC cells (Fig. 1F, Additional File 2: Supplemental Fig. 1O). Collectively, these results suggest a particularly relevant role of NEK2 in TNBC.

### Genome-wide modulation of the TNBC transcriptome by NEK2

**Fig. 2:**
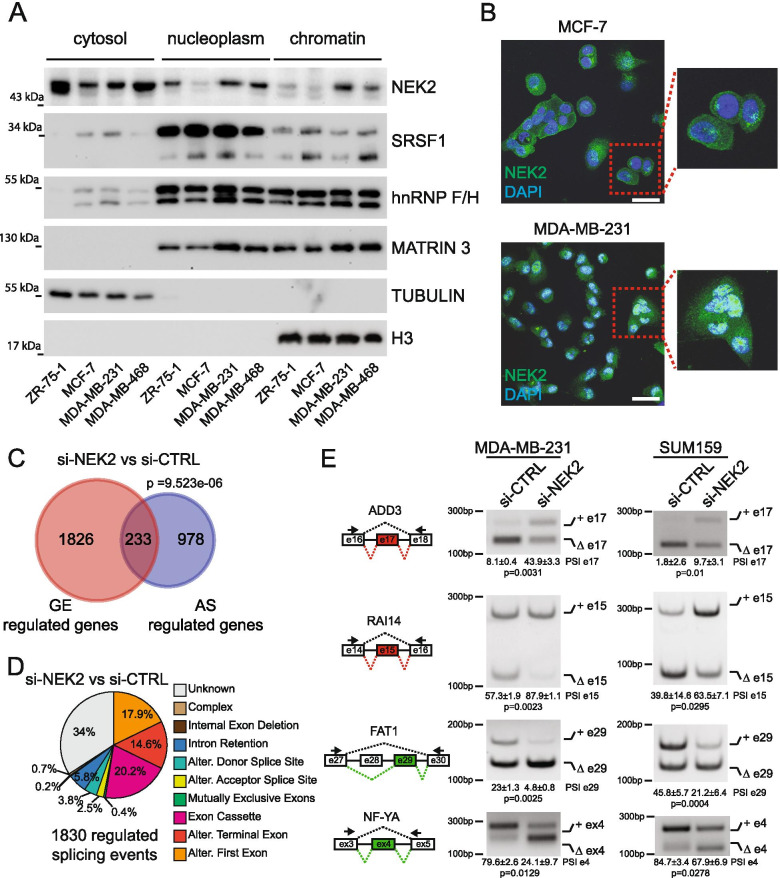
NEK2 regulates TNBC alternative splicing profile. **A** Western blot analysis for NEK2 and splicing factors SRSF1 and hnRNPF/H in cytosolic, nucleoplasmatic and chromatin extracts of BC cell lines. TUBULIN, MATR3 and H3 expression were evaluated as loading control of indicated fraction. **B** Representative images of confocal immunofluorescence analysis using an anti-NEK2 antibody in MCF-7 and MDA-MB-231 cells. DAPI staining was used to identify nuclear morphology. Insets show magnified images. Images were taken using a 60x objective lens. Scale bar 40 μm. **C** Venn diagram showing a significant overlap (hypergeometric distribution test) between genes regulated at the gene expression (GE) and alternative splicing (AS) level in the comparison between NEK2 silenced (si-NEK2) and control (si-CTRL) MDA-MB-231 cells. **D** Pie chart showing percentages of indicated different splicing pattern among regulated splicing events in the si-NEK2 vs si-CTRL comparison. **E** Representative PCR analysis for indicated alternative splicing events in si-CTRL or si-NEK2 MDA-MB-231 (left panel) and SUM159 (right panel) cells. Schematic representation for each event analysed is depicted beside relative agarose gels. Green and red boxes indicate down- and up-regulated exons in si-NEK2 vs si-CTRL cells. Percentage of splicing inclusion (PSI) of indicated exons was evaluated by densitometric analysis, and results are shown below agarose gels (mean ± SD, *n* = 3, t-test)

NEK2 up-regulation in cancer cells was previously associated with its localization in the nucleus [22, 25, 26]. Thus, we analysed the subcellular distribution of NEK2 in BC lines displaying different expression of the kinase. To this end we selected MCF-7, ZR-75-1 as ER^+^/PR^+^ BC cells and MDA-MB-468 and MDA-MB-231 as representative of different basal subtypes within the heterogeneous group of TNBC cells [41]. Fractionation experiments indicated that, in addition to the expected localization in the cytoplasm, a substantial fraction of NEK2 is present in the nucleoplasm and chromatin compartments of TNBC cell lines (MDA-MB-231, MDA-MB-468), where splicing factors like SRSF1 and the heterogeneous nuclear ribonucleoproteins F and H (hnRNP F/H) are also localized (Fig. 2A). By contrast, NEK2 was predominantly localized in the cytoplasm of ER^+^/PR^+^ BC cells (MCF-7, ZR-75-1) (Fig. 2A). Immunofluorescence analysis confirmed the higher enrichment of NEK2 in the nuclear compartment of MDA-MB-231 cells with respect to MCF-7 cells (Fig. 2B, Additional File 2: Supplemental Fig. 2). These observations suggest that the increased nuclear localization of NEK2 in TNBC cells may favour its splicing regulatory activity.

To investigate whether NEK2 expression modulates the transcriptome of TNBC cells, we knocked down its expression in MDA-MB-231 (Additional File 2: Supplemental Fig. 3A), a cell line representative of aggressive TNBC. Paired-end, high-throughput sequencing analysis of polyA-enriched RNA uncovered an extensive modulation of the transcriptome of MDA-MB-231 cells by NEK2. Comparative bioinformatics analyses identified 2059 genes regulated at the expression level and 1211 genes regulated at splicing level, with a significant but limited overlap between the two groups (Fig. 2C and Additional File 3: Supplemental Tables 3–5). These results suggest a multilayered impact of NEK2 on the transcriptome of TNBC cells. Among the 1830 AS events that are susceptible to NEK2 expression, exon cassettes (20.2%) and terminal exons (14.6%) were the most represented splicing patterns (Fig. 2D). We also detected a large fraction of alternative first exon events (17.9%), which likely reflect differential promoter choice. Together with the large number of expression-regulated genes, this finding suggests that NEK2 may affect the expression or activity of key transcription factors in TNBC cells. Given the emerging role of splicing dysregulation in TNBC, we focused on the effects of NEK2 on AS. Analysis by RT-PCR of 14 events confirmed the splicing changes identified by RNA-seq in MDA-MB-231 cells (Fig. 2E and Additional File 2: Supplemental Fig. 3A,C). Furthermore, most of them (~ 86%; *n* = 14) were also susceptible to NEK2 depletion in another TNBC cell line (SUM159; Fig. 2E, Additional File 2: Supplemental Fig. 3B,C). These observations strongly suggest that NEK2 expression impacts on the transcriptome of TNBC cells by modulating AS regulation.

### NEK2 contributes to the TNBC-specific splicing signature

**Fig. 3:**
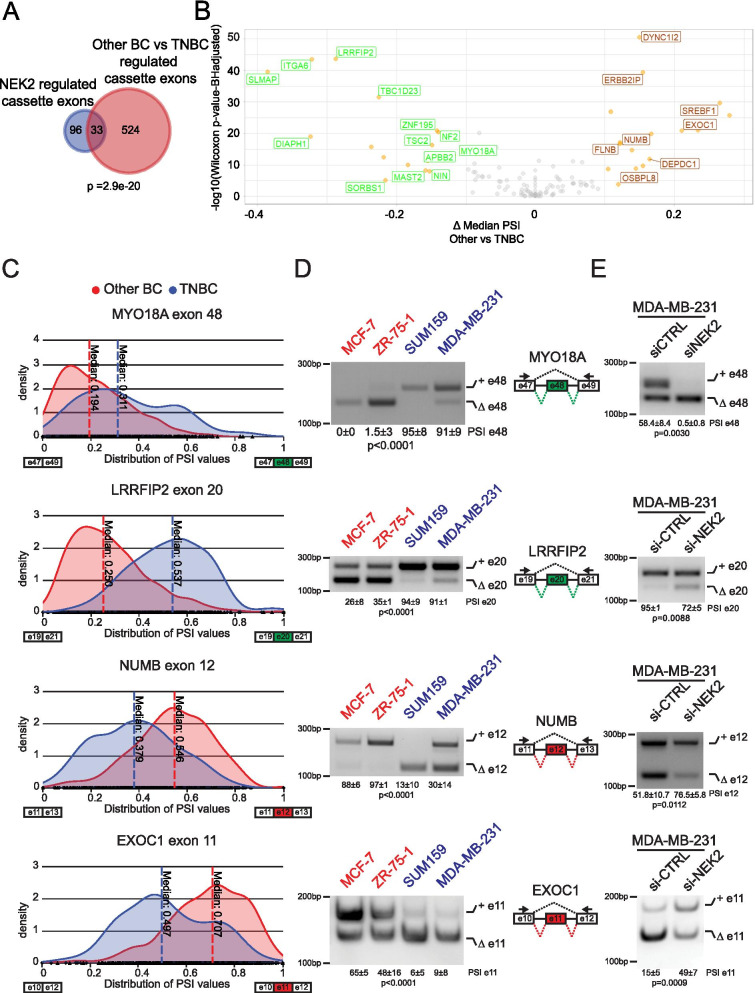
NEK2 regulates TNBC specific splicing events. **A** Venn diagram showing a significant overlap (hypergeometric distribution test) between exon cassettes regulated in the comparison between NEK2 silenced (si-NEK2) and control (si-CTRL) MDA-MB-231 cells and between groups of TNBC and “Other BC” primary tumors. **B** Volcano plot of differential splicing analysis performed for NEK2-regulated cassette exons in MDA-MB-231 between TNBC and “Other BC”. Significantly differentially events are highlighted in orange (|Δ median PSI| ≥ 0.1 and FDR ≤ 0.01, Wilcoxon rank-sum test with Benjamini–Hochberg (FDR) adjustment). Green or red labels indicate down- and up-regulated exons by NEK2 silencing. **C** Density plots showing distribution of the inclusion levels of indicated cassette exons in TNBC and “Other BC”. Analysis of TGCA data in (**A-C**) were performed by using the visual interface of the psichomics R package. **D,E** Representative PCR analysis for indicated alternative splicing events in indicated BC cell lines representative of “Other BC” (ZR-751, MCF-7) or TNBC (MDA-MB-231, SUM159) subtypes (**D**) and in si-CTRL or si-NEK2 MDA-MB-231(**E**). Schematic representation for each event analysed is depicted beside relative agarose gels. Green and red boxes indicate down- and up-regulated exons in si-NEK2 vs si-CTRL cells. Percentage of splicing inclusion (PSI) of indicated exons was evaluated by densitometric analysis, and results are shown below agarose gels [mean ± SD, *n* = 3, one-way ANOVA (**D**), t-test (**E**)]

Next, we asked whether the NEK2-modulated AS events contribute to the splicing signature that discriminates TNBC primary tumors from other BC subtypes. Analysis of NEK2-regulated alternative events annotated as known “pattern” in our reference FAST DB database indicated that the frequency of cassette exons largely exceed that expected from their representation in the genome (Additional File 2: Supplemental Fig. 4A). Moreover, analysis of the TCGA dataset using the psichomics R package [31] pointed to exon cassette as the most represented splicing-pattern among the AS events that are differentially regulated between TNBC and other BC (Additional File 2: Supplemental Fig. 4B; Additional File 4: Supplemental Table 6). Thus, we focused on the exon cassette pattern and further selected 139 events that were commonly annotated in our reference FAST DB database and in psichomics. Interestingly, we found a significant overlap between NEK2-sensitive cassette exons and those differentially spliced between TNBC and the “Other BC” group (Fig. 3A, Additional File 4: Supplemental Table 7). In most cases, NEK2 promoted the TNBC-specific variant and its silencing in MDA-MB-231 reverted the splicing pattern to that observed in the “Other BC” group of patients (Fig. 3B). RT-PCR analysis on representative cell lines of other BC (MCF-7, ZR-75-1) and TNBC (SUM159, MDA-MB-231) subtypes confirmed the differential pattern of inclusion of these cassette exons (Fig. 3C,D). Moreover, depletion of NEK2 in five different TNBC cell lines consistently promoted the splicing pattern observed in the “Other BC” patients and cells (Fig. 3E; Additional File 2: Supplemental Fig. 2C and Supplemental Fig. 4C). Collectively, these observations suggest that up-regulation of NEK2 expression in TNBC contributes to establish their specific splicing signature.

### NEK2 regulates weak cassette exons flanked by low-GC introns

**Fig. 4:**
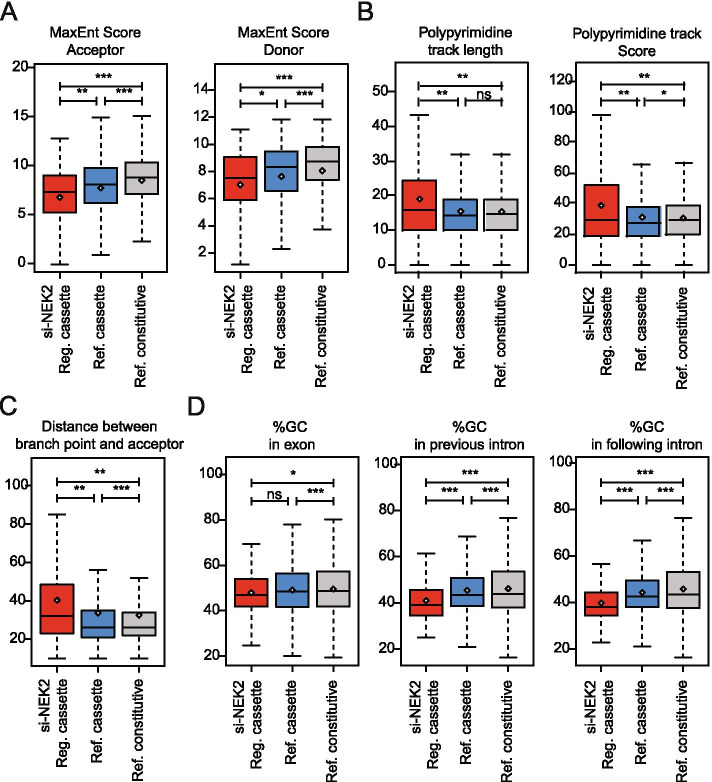
Specific sequence features characterize NEK2 regulated cassette exons. **A** Boxplots showing comparison between NEK2-regulated cassette exons (si-NEK2 reg.cassette, *n* = 139) and other not-regulated cassette exons (ref. cassette, *n* = 11,051) or constitutive exon (ref. constitutive, *n* = 22,480) for strength of their acceptor (right) and donor (left) splice-site. **B** Boxplots showing comparison between indicated groups of cassette exons for the length (left panel) and score of their polypyrimidine tracks (right panel). **C** Boxplot showing distance of the branchpoint from the acceptor splice-site of the indicated groups of cassette exons. **D** Boxplots showing the GC content of indicated groups of cassette exons (left panel) and of their flanking intronic sequences (middle and right panel). Whiskers indicate 1.5 interquartile range and highlighted circle mean values (*p ≤ 0.05, **p ≤ 0.01, ***p ≤ 0.001;ns = not significant, t-test)

To investigate the mechanism of splicing regulation by NEK2, we first explored the structural and sequence features of the regulated cassette exons. NEK2-sensitive exons are characterized by particularly weak 5′ and 3′ splice sites when compared to both reference alternative and constitutive exons (Fig. 4A). Furthermore, they display a longer and stronger polypyrimidine tract compared to both cassette and constitutive exons (Fig. 4B), with a consequent higher distance between the branch-point and the 3′ acceptor splice site (Fig. 4C). These latter features were shown to be enriched in particularly weak exons that have a higher tendency to be skipped [36]. Like other reference cassette exons, NEK2-regulated exons are characterized by a lower GC content compared to constitutively spliced exons (Fig. 4D). However, they are also flanked by introns that have lower GC content even when compared to other cassette exons (Fig. 4D). Interestingly, a lower GC content in both exonic and flanking intronic sequences was recently shown to be specific feature for alternative exons whose inclusion is dependent on U2 snRNP-related proteins [42]. These analyses suggest that NEK2 exerts its splicing effects by regulating the expression or activity of specific RNA binding proteins (RBPs) that act as activator or repressor of weak exons flanked by introns with low GC content.

### RBFOX2 binding motif are enriched within NEK2 sensitive cassette exons

**Fig. 5:**
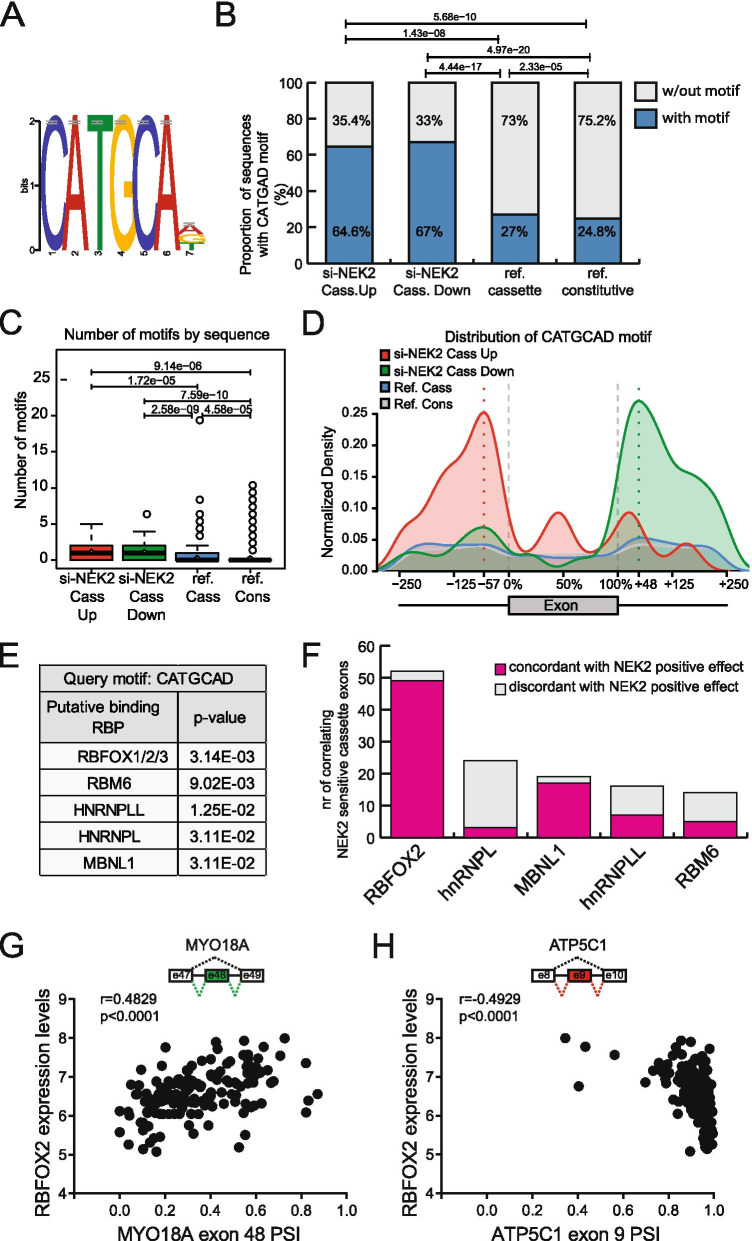
A CATGCAD motif is enriched in NEK2 regulated cassette exons. **A** Logos representing enriched CATGCAD motif within NEK2 regulated cassette exons and flanking introns retrieved from comparative sequence analysis with reference cassette and constitutive exons. **B** Bar graph showing percentage of sequences having the CATGCAD motif in the indicated groups of si-NEK2-up-regulated (*n* = 48), si-NEK2-down-regulated (*n* = 91) cassette exons, reference cassette and constitutive exons. *p* values above the graph indicate a significant difference in the proportion of sequences among indicated groups (χ^2^ test). **C** Box plot showing number of CATGCAD motifs identified in indicated groups of of si-NEK2-up regulated, si-NEK2-down-regulated cassette exons, reference cassette and constitutive exons. p values above the graph indicate a significant difference in the number of motifs among indicated groups (t-test). **D** Curve graph showing distribution of the CATGCAD motif within exons and their flanking intronic sequences (up to 250 nt) in the groups of si-NEK2 up- (red line) and down-regulated exons (green line), reference cassette (blue line) and constitutive (grey line) exons. **E** Table showing results of the search with the Tomtom motif comparison tool of putative RNA-binding proteins cognate to the CATGCAD motif. **F** Bar graph showing the number of NEK2-sensitive cassette exons whose inclusion levels in primary TNBC correlates with the expression of indicated RBPs, in either a concordant or discordant manner with the hypothesis of a positive effect of NEK2 on their activity. **G, H** Scatterplots of PSI values for indicated cassette exons versus normalized expression of RBFOX2 across TNBC patient in TCGA. Spearman’s correlation coefficients (r) and associated *p*-values are shown

To identify RBPs possibly involved in the splicing modulatory function of NEK2, we searched for RNA motifs that are enriched in the NEK2-regulated exons and flanking introns compared to both non-regulated cassette exons and constitutive exons. Five sequence motifs were found to be significantly enriched in the genomic region of NEK2-regulated exons (Additional File 2: Supplemental Fig. 5A), with CATGCAD being the most significant and most abundant sequence element (Fig. 5A). The CATGCAD motif was present in significantly higher proportion in the sequences flanking both up- (64,6%) and down-regulated (67%) exons (Fig. 5B). Furthermore, both types of exons were also characterized by a higher number of motifs with respect to reference cassette exons and constitutive exons (Fig. 5C). However, the distribution of this motif was different in exons regulated in opposite manner, with its enrichment being localized downstream of NEK2-downregulated exons and upstream or within NEK2-upregulated exons (Fig. 5D). This pattern is suggestive of a positional regulatory effect of the cognate splicing factor.

Next, by using the Tomtom comparison tool, we screened a compendium of known RBPs for their potential affinity for the CATGCAD motif [37]. Notably, the CATGCAD motif displayed the highest similarity to the binding sites for splicing factors that are known to act in a position-dependent manner, such as RBFOX1–3, RNA binding motif protein 6 (RBM6), hnRNPL and Muscleblind like splicing regulator 1 (MBNL1) (Fig. 5E) [43–46]. Among the RBFOX proteins, which showed the most significant potential for binding to the motif (Fig. 5E), we focused our attention on RBFOX2, as transcriptomic data from the CCLE project [35] revealed barely detectable expression levels for RBFOX1 and 3 both in our reference model, MDA-MB-231 cells, and in other ER^−^/HER2^−^ BC cell lines (Additional File 2: Supplemental Fig. 5B). Interestingly, RBFOX2 expression levels also correlated with the highest number of NEK2-regulated exons in the transcriptome of TNBC patients (Fig. 5F) and, for most of them (94.2%), in a manner that is coherent with a positive effect of NEK2 on RBFOX2 function. Indeed, events that positively correlated with RBFOX2 expression in patients, were downregulated in NEK2-depleted MDA-MB-231 cells (i.e. exon 48 of MYO18A; Fig. 5G), whereas those negatively correlating with RBFOX2 in patients were up-regulated in NEK2-depleted MDA-MB-231 cells (i.e. ATP5C1; Fig. 5H). Since RBFOX2 is known to promote exon inclusion when binding in the downstream intron and to repress it when binding in the upstream intron or within the exon [44], these analyses are in line with the hypothesis of an impairment of its splicing activity upon depletion of NEK2.

### NEK2 modulates an RBFOX2-dependent pro-mesenchymal splicing program in TNBC cells

**Fig. 6:**
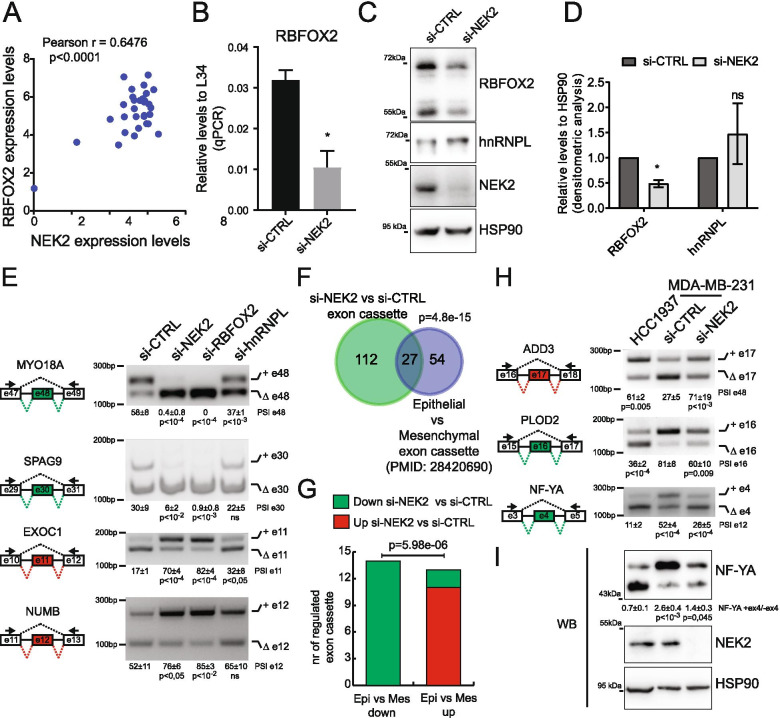
NEK2 modulates an RBFOX2-dependent pro-mesenchymal splicing program in TNBC cells. **A** Scatter plot of RNA expression levels of *RBFOX2* and *NEK2* across multiple ER^−^/HER2^−^ BC cell lines according to data of CCLE project. Pearson’s correlation coefficient (r) and associated *p*-value are shown. **B** qRT-PCR analysis of relative expression levels of RBFOX2 to L34 in control or NEK2-silenced MDA-MB-231 cells (mean ± SD, *n* = 3, *p-value≤0.05 t-test). **C,D** Western Blot (**C**) and densitometric analysis (**D**) of expression levels of RBFOX2, hnRNPL and NEK2 in si-CTRL or si-NEK2 MDA-MB-231 cells. HSP90 was evaluated as loading control (mean ± SD, *n* = 3, levels in si-CTRL were set to 1, *p-value≤0.05, ns = not significant, t-test). **E**) PCR and densitometric analysis of percentage of splicing inclusion (PSI) for indicated alternative splicing events in MDA-MB-231 cell transfected with control or NEK2, RBFOX2, hnRNPL targeting siRNAs (mean ± SD, *n* = 3, one-way ANOVA). Schematic representation for each event analysed is depicted beside relative agarose gels. Green and red boxes indicate down- and up-regulated exons in si-NEK2 vs si-CTRL cells. **F,G** Venn diagram showing alternative exon-cassettes overlapping (hypergeometric distribution test) in the comparison between control and NEK2-silenced cells and between epithelial and mesenchymal breast cancer cells from indicated study (**F**) and bar graph showing their regulation in the two comparison (Fisher’s exact test) (**G**). **H** PCR and densitometric analysis of PSI for indicated EMT-related cassette exons in HCC1937 and control or NEK2-silenced MDA-MB-231 cells (mean ± SD, *n* = 3, one-way ANOVA). Schematic representation for each event analysed is depicted beside relative agarose gels ad in (**E**). **I** Western Blot and densitometric analysis of the ratio between expression levels of NF-YA isoforms including or excluding exon 4 analysis for NF-YA in HCC1937 and control or NEK2-silenced MDA-MB-231 cells (mean ± SD, *n* = 4, one-way ANOVA). NEK2 and HSP90 were evaluated as control

Analysis of the RNA-seq data for gene expression changes indicated that RBFOX2 expression was the only CATGCAD motif-binding RBP regulated in NEK2-silenced cells (Additional File 2: Supplemental Fig. 6A). Moreover, computational analysis of datasets from multiple ER^−^/HER2^−^ BC cell lines revealed a significant correlation between the expression levels of NEK2 and RBFOX2 (Fig. 6A). Accordingly, RBFOX2 was significantly down-regulated at both transcript and protein level in NEK2-depleted MDA-MB-231 cells (Fig. 6B-D). RBFOX2 downregulation in NEK2-depleted cells was not determined by reduced transcript stability, as analysis of its expression after transcription inhibition indicated no changes in the decay of RBFOX2 transcript, which is relatively stable with respect to a high turnover transcript like MYC mRNA (Additional File 2: Supplemental Fig. 6B). This observation suggests that NEK2 knockdown represses transcription of RBFOX2 mRNA. Notably, this effect appears specific, as no significant changes were observed for hnRNPL expression (Fig. 6C,D), which was selected as the other RBP correlating with a high proportion of NEK2-regulated exons (Fig. 5F).

To test the hypothesis that RBFOX2 modulates the splicing of NEK2-sensitive exons, we silenced its expression in MDA-MB-231 cells. With the only exception of *SPTAN1* exon 24 (Additional File 2: Supplemental Fig. 7A), all other arbitrarily selected cassette exons were modulated in the same direction by either RBFOX2 or NEK2 depletion, in most cases with a greater fold change when RBFOX2 was knocked down (Fig. 6E; Additional File 2: Supplemental Fig. 7A). On the other hand, depletion of hnRNPL mildly affected only few events (Fig. 6E; Additional File 2: Supplemental Fig. 7A). Importantly, NEK2 positively regulated the expression of RBFOX2 in another mesenchymal TNBC cell line (MDA-MB-436) and NEK2-dependent splicing events were susceptible to RBFOX2 depletion also in these cells (Additional File 2: Supplemental Fig. 7B-D).

The splicing activity of RBFOX2 was shown to drive splicing changes during epithelial to mesenchymal transition (EMT) [47] and to promote a pro-mesenchymal program in BC cells [48]. Remarkably, we observed a highly significant overlap between cassette exons regulated by NEK2 and those differentially expressed between epithelial and mesenchymal BC cells and tissues identified in a previous study [49] (Fig. 6F, Additional File 4: Supplemental Table 8). This comparative analysis also revealed that NEK2 depletion inhibits the mesenchymal-enriched pattern in most of these 27 splicing events (92,5%; Fig. 6G). Moreover, comparison of arbitrarily selected EMT-related events between epithelial (HCC1937) and mesenchymal (MDA-MB-231) TNBC cells confirmed the differential inclusion of these exons and the effect of NEK2 on promoting the mesenchymal splicing pattern (Fig. 6H and Additional File 2: Supplemental Fig. 7E). Furthermore, Western blot analysis confirmed the increased expression of the epithelial-enriched short isoform of the NF-YA transcription factor in NEK2-depleted mesenchymal TNBC cells (Fig. 6I), thus validating the splicing switch at protein level. These results indicate that NEK2 promotes a pro-mesenchymal splicing pattern in TNBC cells through regulation of RBFOX2 expression and activity.

### NEK2-induced splice variants promote a mesenchymal phenotype in TNBC cells

**Fig. 7:**
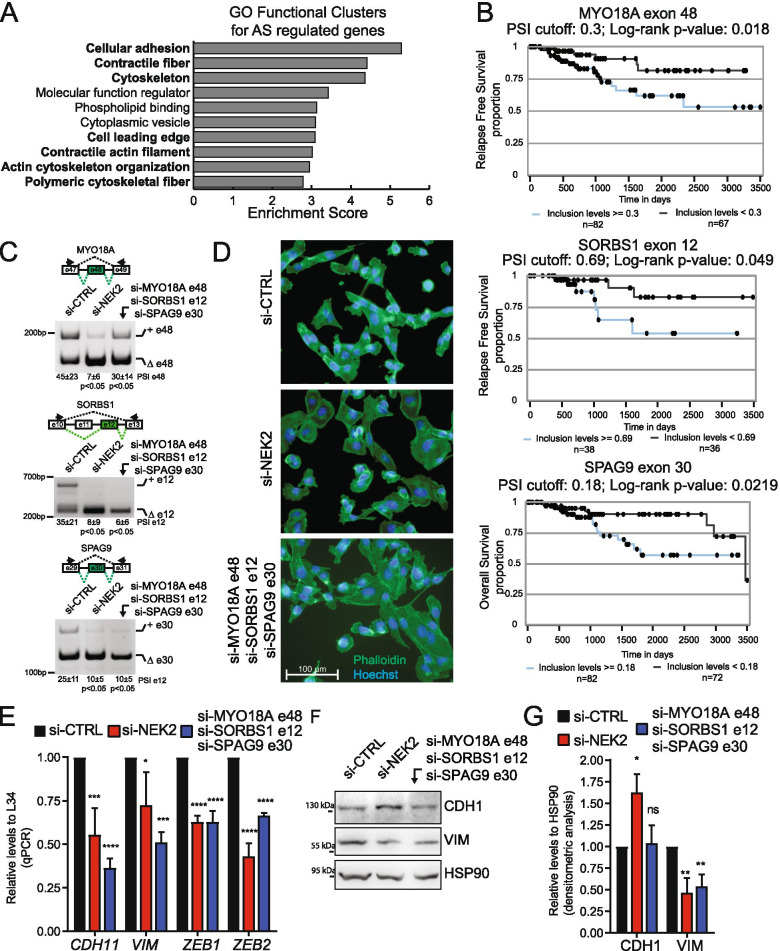
NEK2-induced splice variants promote a mesenchymal phenotype in TNBC cells. **A** Bar graphs showing the enrichment score of the top 10 gene ontology (GO) functional clusters enriched in AS regulated genes in the comparison between control and NEK2 silenced MDA-MB-231 cells (fold enrichment ≥2.0, *p*-value ≤0.05). GO-clusters related to cell migration and cytoskeletal remodeling are highlighted in bold. **B** Kaplan–Meier survival curves illustrating the relapse free survival or overall survival probability of TNBC patients stratified according to inclusion levels of indicated NEK2-sensitive cassette exons. PSI-cut-off and *p*-value are indicated above each graph. Analysis and graphs were drawn by visual interface of the psichomics R package. **C** PCR analysis evaluating inclusion levels of indicated cassette exons of *MYO18A*, *SORBS1* and *SPAG9* genes in MDA-MB-231 cells transfected with siRNAs targeting either NEK2 or indicated specific cassette exons compared to control. Schematic representation for each event analysed is depicted besides relative agarose gels. Percentage of splicing inclusion (PSI) of indicated exons was evaluated by densitometric analysis, and results are shown below agarose gels (mean ± SD, *n* = 6, t-test). **D** Representative micrographs of MDA-MB-231 cells cytoskeleton stained with FITC- phalloidin upon transfection with indicated siRNAs. Hoechst dye counterstains nuclei. **E** qRT-PCR analysis of expression levels of *CDH11, VIM, ZEB1* and *ZEB2* relative to L34 in MDA-MB-231 cells transfected with siRNAs targeting either NEK2 or indicated specific cassette exons compared to control. (mean ± SD, *n* = 4, control value set to 1, *p ≤ 0.05, **p ≤ 0.01, ****p ≤ 0.0001, one-way Anova). **F, G** Western Blot (**F**) and densitometric analysis (**G**) of expression levels of E-cadherin (CDH1) and vimentin (VIM) protein in MDA-MB-231 cells transfected with indicated siRNAs. HSP90 was evaluated as loading control (mean ± SD, *n* = 3, levels in si-CTRL were set to 1, *p-value≤0.05,** ≤0.01; ns = not significant, one-way Anova)

**Fig. 8:**
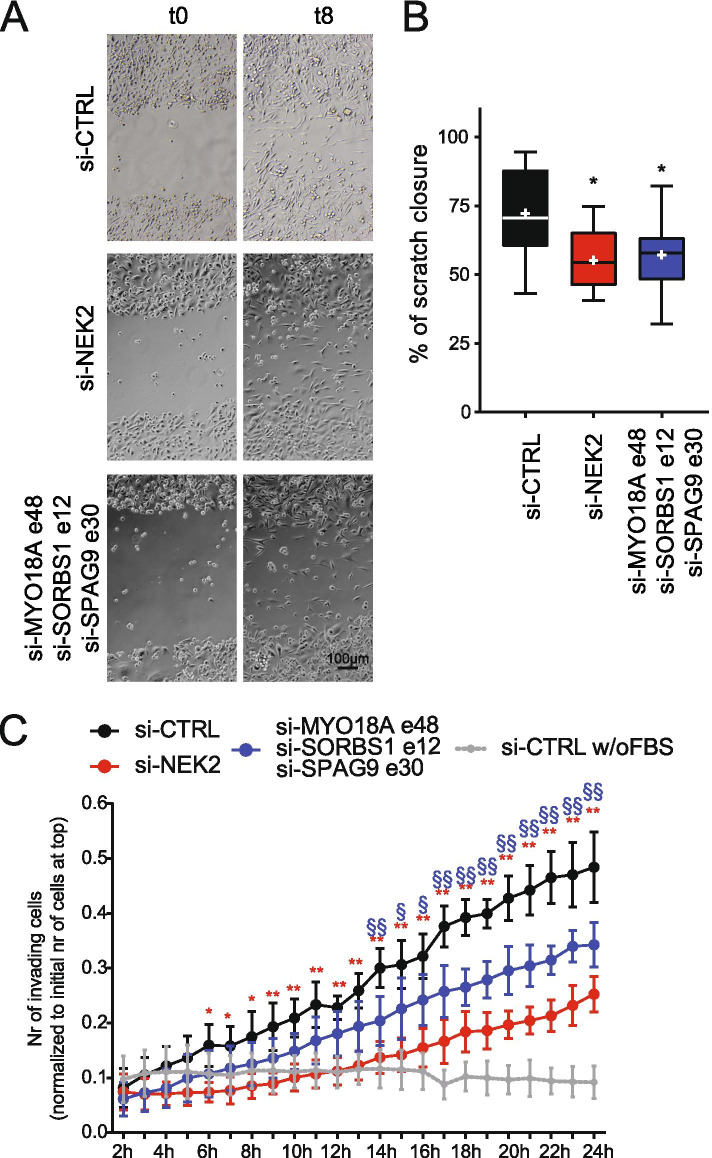
NEK2-induced splice variants regulate migratory properties of TNBC cells. **A** Representative images of the scratch assay performed on confluent layers of MDA-MB-231 cells transfected with indicated siRNAs, at the time scratch was produced (t0) and 8 h later (t8). **B** Boxplot showing results of quantification of scratch assay displayed in (**A**). Whiskers indicate min and max value. + mark mean values (*n* = 3, for each biological replicate, at least 3 measurements were made, one-way Anova). **C** Line graph showing number of MDA-MB-231 cells, transfected with indicated siRNAs, invading Matrigel-coated transwell of the IncuCyte Clearview 96well insert system. Cell invasion was monitored for 24 h post-seeding using the IncuCyte® SX5 Live-Cell Analysis Imaging System. Migration of control MDA-MB-231 cells towards a lower chamber filled with medium without FBS (w/o FBS) is illustrated as negative control. Number of invading cells on the bottom side of the insert at every hour was normalized to the initial number of cells on the top of the insert at initial seeding (mean ± SD, *n* = 6, *p ≤ 0.01, **p ≤ 0.001, two-way Anova)

Functional annotation of the genes regulated by NEK2 at the splicing level revealed a significant enrichment of terms relative to cellular adhesion and contractile cytoskeleton (Fig. 7A). These processes are extensively modulated during EMT in cancer cells and promote progression toward a metastatic stage of disease [50]. Notably, dysregulation of EMT characterizes TNBC with an aggressive and metastatic phenotype [2, 3]. Thus, we asked if the inclusion levels of NEK2-regulated cassette exons could segregate TNBC patients with different clinical outcome. Analysis of the available data regarding relapse-free survival (RFS) or overall survival (OS) probability from the TCGA identified 24 NEK2-sensitive cassette exons that were prognostic in TNBC patients and for which NEK2 promotes the splicing pattern correlated with worse prognosis (Fig. 7B, Additional File 2: Supplemental Fig. 8A). Moreover, we observed a highly significant overlap between NEK2-regulated exons and the minimal splicing signature that identifies basal-B-like patients [51] (Additional File 2: Supplemental Fig. 8B). These computational analyses suggest that the splicing program modulated by NEK2 is oncogenic in TNBC cells. To further corroborate this hypothesis, we investigated the functional consequences of modulating the expression levels of specific splice-isoforms regulated by NEK2: *MYO18A* exon 48^+^, *SORBS1* exon 12^+^, *SPAG9* exon 30^+^. Inclusion of these three exons is promoted by NEK2 in TNBC cells (Fig. 7C) and correlates with worse prognosis in TNBC patients (Fig. 7B). Transfection of exon-specific siRNAs was able to specifically down-regulate the NEK2-induced splice variant without affecting the alternative isoform of these genes (Fig. 7C; Additional File 2: Supplemental Fig. 8C). Phalloidin-staining of the cytoskeleton revealed substantial morphological changes in MDA-MB-231 cells upon knockdown of NEK2, with loss of the typical elongated shape of mesenchymal cells and gaining of a cuboidal, epithelial-like phenotype (Fig. 7D). Remarkably, the same effect was achieved by concomitantly targeting the NEK2-induced splice variants of the *MYO18A*, *SORBS1* and *SPAG9* genes (Fig. 7D). Consistently with the morphological changes observed, qRT-PCR analysis revealed the downregulation of transcripts encoding mesenchymal markers associated with poor prognosis in TNBC, such as the membrane protein cadherin 11 (CDH11*)* [52], the cytoskeletal vimentin protein (VIM) and the two key transcription factors zinc finger E-box binding homeobox 1 (ZEB1) and 2 (ZEB2) [53] (Fig. 7E). Reduced vimentin expression was also assessed at the protein level by Western blot analysis in MDA-MB-231 cells depleted for either NEK2 or its target splice-variants, while a slight increase in the levels of the epithelial marker E-cadherin (CDH1) was only observed in NEK2-silenced cells (Fig. 7G). These observations support the functional involvement of NEK2-susceptible splice-variants of the *MYO18A*, *SORBS1* and *SPAG9* genes in the pro-mesenchymal program promoted by this kinase. Moreover, knockdown of either NEK2 or the three “mesenchymal” splice variants impaired the migratory properties of MDA-MB-231 cells, as shown by results of wound-healing and Matrigel-invasion assays (Fig. 8A-D). Collectively, these observations suggest that NEK2 promotes the migratory and invasive phenotype of TNBC cells by orchestrating a pro-mesenchymal splicing program.

## Discussion

TNBC displays the poorest prognosis among the BC subtypes, mainly because of its higher rate of recurrence and metastasis and increased likelihood of developing drug resistance [2, 3]. Moreover, lack of TNBC-specific targeted therapies contributes to the negative outcome of the disease. Thus, identification of therapeutically actionable targets represents a clinical priority for the improvement of TNBC management. In this regard, mounting evidence suggests that the mitotic kinase NEK2 plays a key oncogenic function in BC [19, 20, 23]. However, while most studies have focused on its role in the cytoplasm as a regulator of cell division, we and others have shown that NEK2 also localizes in the nucleus of cancer cells, including BC cells, where it regulates splicing of cancer-relevant genes [26, 27]. Herein, by querying transcriptomic data of large cohorts of BC patients from the multiple transcriptomics projects, we describe that NEK2 is significantly upregulated in TNBC with respect to other BC subtypes and to normal mammary tissue. Moreover, our results document that NEK2 is specifically increased in the nuclear and chromatin-bound fractions of TNBC cells and that its depletion exerts a widespread effect on the transcriptome of TNBC cells, by modulating thousands of genes at transcriptional and/or splicing level. This genome-wide regulation of nuclear RNA processing events by NEK2 is similar to what recently reported for another oncogenic mitotic kinase, AURKA. Indeed, up-regulation of AURKA in cancer cells also promotes its translocation to the nucleus, where it regulates the activity of transcription and splicing factors, resulting in modulation of gene expression programs [54]. Thus, it is tempting to speculate that the upregulation of mitotic kinases, including NEK2 and AURKA, frequently observed in many cancer types results in the gain of a nuclear function that directly or indirectly modulates the transcriptome of cancer cells.

Recent studies have indicated that AS signatures exhibit a higher proficiency than gene expression signatures in discriminating TNBC from other BC subtypes [11–13]. Although TNBC is highly heterogeneous, this splicing signature represents a common feature that characterize all these tumors. These observations suggest that TNBC-specific splice variants might functionally contribute to their generally negative prognosis. On this basis, we focused our study on the effects elicited by NEK2 on AS regulation in TNBC cells. Importantly, several NEK2-regulated cassette exons identified in our study are also differentially included in primary TNBCs compared to other BCs. Moreover, we confirmed the NEK2-dependent regulation of these exons in multiple cell lines that are representative of different TNBC subtypes. Collectively, these findings support a key role of NEK2 in the establishment of the TNBC-specific splicing signature and suggest that interference with NEK2 expression may represent an efficacious tool to interfere with the AS program characterizing TNBCs.

Bioinformatics analyses of the structural features of NEK2-regulated exons highlighted their particularly weak nature. These exons harbor splice sites that are significantly weaker than other non-regulated cassette exons and are flanked by introns characterized by low GC content. These features suggested that NEK2 may promote the activity or expression of auxiliary splicing factors that assist the spliceosome in the recognition of weak cassette exons. To identify such putative splicing factors, we searched for RNA binding motifs that are enriched in the regulated exons and flanking introns. Computational analyses predicted several sequence motifs, with the CATGCAD sequence being the most significantly enriched one in NEK2-regulated exons. This sequence motif is closely related to the binding site for the RBFOX family and our functional analyses revealed that NEK2 mainly elicits its effects by promoting the expression of RBFOX2. Coherently with the known positional activity of RBFOX2 [44], we observed an enrichment of CATGCAD motif upstream of NEK2-repressed exons and downstream of the NEK2-induced exons. Notably, the splicing activity of RBFOX2 was shown to be concentration dependent. When RBFOX2 is lowly expressed, it preferentially regulates transcripts with high affinity motifs, whereas at high expression levels it can also act on transcripts harboring multiple low affinity binding sites [55]. The close resemblance of NEK2-enriched motif with the RBFOX2 primary binding site (GCAYG), and the correlation between expression of this splicing factor and the inclusion of NEK2-regulated exons in TNBC patients, suggest that NEK2 sustains the minimal/optimal levels of RBFOX2 necessary to exert its splicing activity in TNBC cells. Interestingly, RBFOX2 can modulate splicing even in the absence of its direct binding to target RNAs, through the interaction with partner splicing factors that bridge it to the pre-mRNA [56, 57]. One of the most recently identified partners of RBFOX2 is the oncogene SRSF1 [56], whose splicing activity is stimulated by NEK2-dependent phosphorylation [26]. Since SRSF1 is also oncogenic in BC [15], it is conceivable that part of the NEK2 role in TNBC involves the modulation of the functional interaction between RBFOX2 and SRSF1 and consequent genome-wide transcriptome changes.

EMT is a complex developmental process reactivated by cancer cells both at the early stages of their development and at later phase of metastatic spreading or during acquisition of drug resistance [50]. Several studies have highlighted AS reprogramming during EMT in cancer cells. Strikingly, we observed a large overlap of NEK2-regulated exons with those differentially included between epithelial and mesenchymal BCs. This correlation is likely associated with the positive effect of NEK2 on RBFOX2 function. Indeed, RBFOX2 expression increases during the acquisition of a mesenchymal phenotype in BC cells [47, 48]. RBFOX2 was also shown to be a major regulator of EMT [48, 50] and to promote the mesenchymal splicing pattern of several cancer-relevant genes [58]. Furthermore, RBFOX2 binding sites are enriched near alternative exons that characterize BCs displaying mesenchymal traits and highly invasive potential, such as the claudin-low and basal-B subtypes [2, 3, 48, 59]. In line with these observations, we also report that knockdown of NEK2 reduces transcript and protein expression of the long splice variant of the transcription factor NF-YA, which correlates with a mesenchymal and more aggressive phenotype in BC [60]. Remarkably, 9 out of the 25 alternative exons recently identified as the minimal splicing signature that identifies basal-B-like patients with worst prognosis are susceptible to regulation by NEK2 [51]. Collectively, these observations strongly suggest that elevated NEK2 levels in TNBC cells support a pro-mesenchymal splicing program by controlling RBFOX2 expression, thus enhancing tumor aggressiveness and invasiveness. In line with this notion, specific silencing of three NEK2-sensitive splice-variants (MYO18A, SORBS1 and SPAG9), which were selected because they correlate with worse prognosis in TNBC patients, largely mimics NEK2 depletion, by reducing the expression of mesenchymal markers, altering the morphology and reducing the motility of mesenchymal TNBC cells. In this regard, our study provides an example of the great potential of integrating transcriptomics and clinical data for the identification of therapeutic targets in cancer and highlights the remarkable value of splice variants in this context. Indeed, oncogenic splice variants, such as those regulated by NEK2, may represent actionable therapeutic targets that can be selectively suppressed by RNA-based drugs, such as siRNAs or splice-switching antisense oligonucleotides.

## Conclusions

NEK2 modulates an extensive splicing program involving splice variants that confer an invasive and aggressive phenotype to TNBCs. Thus, our study suggest that this kinase may not only represent a valuable therapeutic target for TNBC, but it could also serve as biomarker for the selection of patients that may benefit of personalized, RNA-based therapies. Indeed, since RNA therapeutics is a flourishing field that holds promise for rapid development in the clinical setting [7, 61], further understanding of the mechanistic insights of splicing dysregulation in human cancers may pave the ground for a novel generation of highly selective therapeutic drugs.

## Supplementary Information

**Additional file 1: Supplemental Tables 1–2.** Tables listing sequences of siRNAs and primers used in this study.

**Additional file 2: Supplemental Figures. 1–8.** Supplemental Figures and their figure legends.

**Additional file 3: Supplemental Tables 3–5.** Tables listing results of RNA-seq data analysis.

**Additional file 4: Supplemental Table 6–8.** Tables listing results of comparative analyses of RNA-seq data from this manuscript and data from TCGA and PMID: 28420690.

## Data Availability

RNA-seq data are available on GEO database (accession number GSE140803).

## Abbreviations

TNBC: Triple-negative breast cancer
BC: Breast cancer (BC)
AS: Alternative splicing (AS)
EMT: Epithelial to mesenchymal transition
ER: Estrogen receptor
PR: Progesterone receptor
RNA-seq: RNA sequencing
TCGA: The Cancer Genome Atlas
CCLE: Cancer Cell Line Encyclopedia
RBP: RNA binding protein
