# Supplementary material for: The oncogenic kinase NEK2 regulates an RBFOX2-dependent pro-mesenchymal splicing program in triple-negative breast cancer cells
Source: J Exp Clin Cancer Res. 2021 Dec 20;40:397. doi: 10.1186/s13046-021-02210-3 (PMC8686545; doi:10.1186/s13046-021-02210-3)
Supplement: Supplementary file 1 — Additional file 1: Supplemental Tables 1–2. Tables listing sequences of siRNAs and primers used in this study. [file 13046_2021_2210_MOESM1_ESM.pdf]

Supplemental Table 1. Sequences of si-RNAs used in this study

**si-RNA name**	**siRNA sequence (5'-3')**
siCTRL_1	UGGUUUACAUGUCGACUAA
siCTRL_2	UGGUUUACAUGUUGUGUGA
siCTRL_3	UGGUUUACAUGUUUUCUGA
siCTRL_4	UGGUUUACAUGUUUUCCUA
si-NEK2_1	GGAUCUGGCUAGUGUAAUU
si-NEK2_2	GCAGACAGAUCCUGGGCAU
si-NEK2_3	GGCAAUACUUAGAUGAAGA
si-NEK2_4	GCUAGAAUAUUAAACCAUG
si-RBFOX2_1	CUCACUAUGUUCUUUGAAU
si-RBFOX2_2	GGGUUCGUAACUUUCGAGA
si-RBFOX2_3	AUAUGCAAAUGGUUGGAAA
si-hnRNPL	GCUUGAAUGUGUUCAAGAA
siMYO18A exon 48	AUUCUUUCUUUUCUGAUACUU
siSPAG9 exon 30	UAAACGUCCCUGGUGGAGGAU
siSORBS1 exon 12	AUCUAGAGUCGGGAAGGUCAG

Supplemental Table 2. List of sequences of primers used in this study

Primer name	Sequence (5'-3')
ADD3 FW	ACCAGCTCCTCCTAACCCAT
ADD3 REV	CTACCATGACAGGCACTTCCA
ATP5C1 EX 10 REV	GGACAAAGGCAGCAGTAAGC
ATP5C1 EX 8 FW	CATTCAACCGTACCCGCCAA
CDH11 FW	TGGCAGCAAGTATCCAATGG
CDH11 RV	TTTGGTTACGTGGTAGGCAC
CLSTN1 FW	TGTGACTGAGGATTACCCGCT
CLSTN1 REV	GAACGGAGAGTTAAGCCAGCC
EXOC1 EX 10 FW	TGCAAAGATCAAGATGACTGGCA
EXOC1 EX 12 REV	ACTGAGATCTGCGATTCCCTGA
FAT1 FW	GCCTGTCTGAAGTGCAGTCTC
FAT1 REV	GGGAACTCTTGTATGTCCGGC
FKBP9 FW	GCTTGCCTACGGAAATGAAG
FKBP9 REV	GGGGCTTGAAATAGGTGTGA
GAPDH FW	CCCTTCATTGACCTCAACTACATG
GAPDH REV	TGGGATTTCCATTGATGACAAGC
ITGA6 EX 26 FW	TCGGGAGTACCTTGGTGGATC
ITGA6 EX 28 RV	TTAGGCCCCCGCTATGAGTAG
KIF13A EX 38 FW	GCAAGGCCTCTTCTAAGCCAG
KIF13A EX 40 REV	GCACTGTGGGAAAAGTAGCCA
L34 FW	GTCCCGAACCCCTGGTAATAGA
L34 REV	GGCCCTGCTGACATGTTTCTT
LRRFIP2 EX 22 FW	CATCCAGACGAGGAAGTGGG
LRRFIP2 EX 24 RV	CTGCCATCTGTTCCTCCTGC
MARK3 FW	CATTGTCCCAGACTCGAAGCC
MARK3 REV	GGGATCGAGGCTTTGCTTCTT
MYO18A EX 47 FW	AGTTGGCATTCAAGCGCATC
MYO18A EX 49 REV	GACAACCAGGACTTGACCCC
NEK2 FW	AGGAAATTCAGTTACAGGAGCG
NEK2 RV	TCCTCTGCTAGTCTCTCACGA
NFY-A FW	GGAGCAGTATACAGC
NFY-A REV	GCTGCAAACCCTGTGTTCC
NUMB EX 12 FW	AGGACCCCTTCTCATCTGCT
NUMB EX 13 REV	GCACCAGAAGATTGACCCCA
PLOD2 FW	GCTCTTTGCCGAAATGCTAGA
PLOD2 REV	TTCAAAAATCTGCCAGAGGTCA
RAI14 EX 14 FW	AAGACCCCAACAAAACCAAAGC
RAI14 EX 16 REV	CTTGAAGGGTGGTTCAGCAAA
RBFOX2 FW ex8	GCTACAGCACGTGTAATGACC
RBFOX2 REV ex10	TTGCCTAGGGACACATCTGC
SORBS1 ex 10 FW	GTGGAGGCTTTCTTCCAGTGC
SORBS1 ex 13 REV	CTCGATTGTGTTGCAAGAGC
SPAG9 EX 29 FW	TGTAATCGTTTGTGGGTGGGG
SPAG9 EX 31 REV	CACGGATTACACTTCCAGGACG
SPTAN1 EX 23 FW	GTGAAGAAATTGGACCCCGC
SPTAN1 EX 25 REV	CCTCCTCACTTGTCAGAGCG
SUCO FW	TGGTAGAGATGAGCGGAATGT
SUCO REV	TGGACAAAAGTGCTCTGATCCA
VIM FW	AGACACTATTGGCCGCCTGCAGGATG
VIM RV	CCCTCAGGTTCAGGGAGGAAAA
ZEB1 FW	ACTCAACTACGGTCAGCCCT
ZEB1 RV	TGGGCGGTGTAGAATCAGAG
ZEB2 FW	CAAGAGGCGCAAACAAGCC
ZEB2 RV	GGTTGGCAATACCGTCATCC
