# Supplementary material for: The oncogenic kinase NEK2 regulates an RBFOX2-dependent pro-mesenchymal splicing program in triple-negative breast cancer cells
Source: J Exp Clin Cancer Res. 2021 Dec 20;40:397. doi: 10.1186/s13046-021-02210-3 (PMC8686545; doi:10.1186/s13046-021-02210-3)
Supplement: Supplementary file 2 — Additional file 2: Supplemental Figures. 1–8. Supplemental Figures and their figure legends. [file 13046_2021_2210_MOESM2_ESM.pdf]

Additional File 2. Supplemental figure and figure legends 1-8.

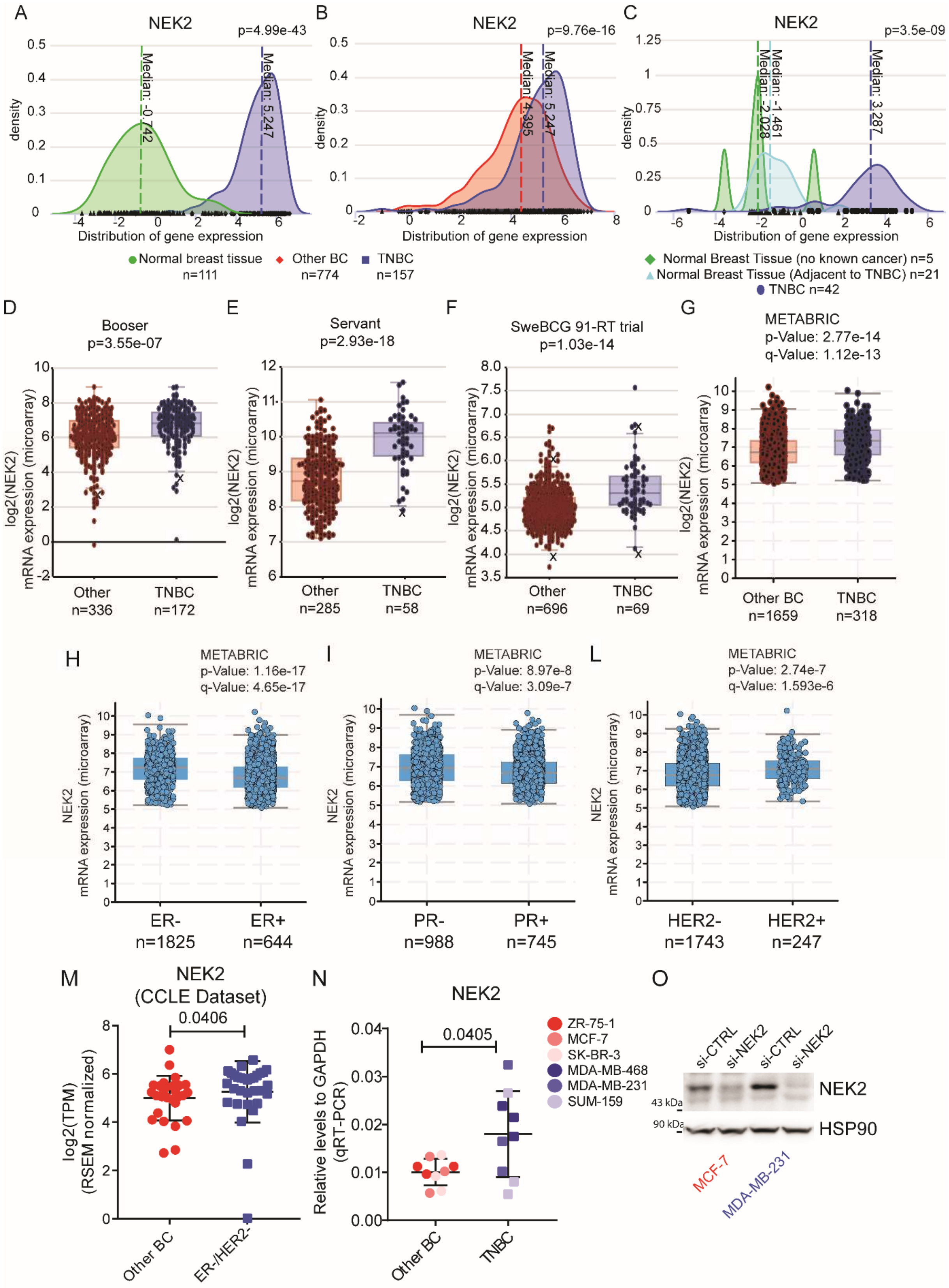

Supplemental Figure 1. NEK2 is highly expressed in triple negative and HER2+ breast cancers. A, B, C) Density plots of NEK2 gene expression distribution in triple-negative (TNBC) breast cancers compared to normal breast tissue (A), other breast cancer subtypes (Other BC) (B) and normal breast tissues, derived by either adjacent regions to the primary tumor (light blue curve line) or from reduction mammoplasty in patients with no known cancer (green curve line) (C). TCGA (A, B) and SRP042620 data (C) were analyzed using psichomics R package visual interface. Indicated p-value in (A, B) were estimated by Wilcoxon rank sum test with continuity correction, in (C) by Kruskal-Wallis rank sum test. D-G) Boxplots showing NEK2 expression levels in TNBC or Other BC according to analysis of indicated microarray datasets. Analyses were performed using R2: Genomics Analysis and Visualization Platform (D-F) or cBIOportal platform (G). H-L) Boxplots showing NEK2 expression levels in BC patients stratified according to positive (+) or negative (-) expression of estrogen (ER) (B), progesterone (PR) (C) and HER2 (D) receptors according to analysis of the METABRIC dataset using the cBIOportal database. M) Boxplot showing NEK2 expression levels in breast cancer cell lines representative of the "Other BC" or ER^-/$HER2^-$ subtypes, according to transcriptomic data from the Cancer Cell Line Encyclopedia (CCLE) project (indicated p-value were estimated with Mann Whitney test). N) Boxplot showing result of qRT-PCR analysis of relative expression levels of NEK2 to GAPDH in indicated cell lines, representative of the "Other BC" or TNBC subtypes (indicated p-value were estimated with Mann Whitney test). O) Representative Western blot analysis assessing NEK2 silencing efficiency in MCF-7 and MDA-MB-231 cells, 48h post transfection with indicated control (si-CTRL) or NEK2-targeting (si-NEK2) pool of siRNAs. HSP90 was evaluated as loading control.

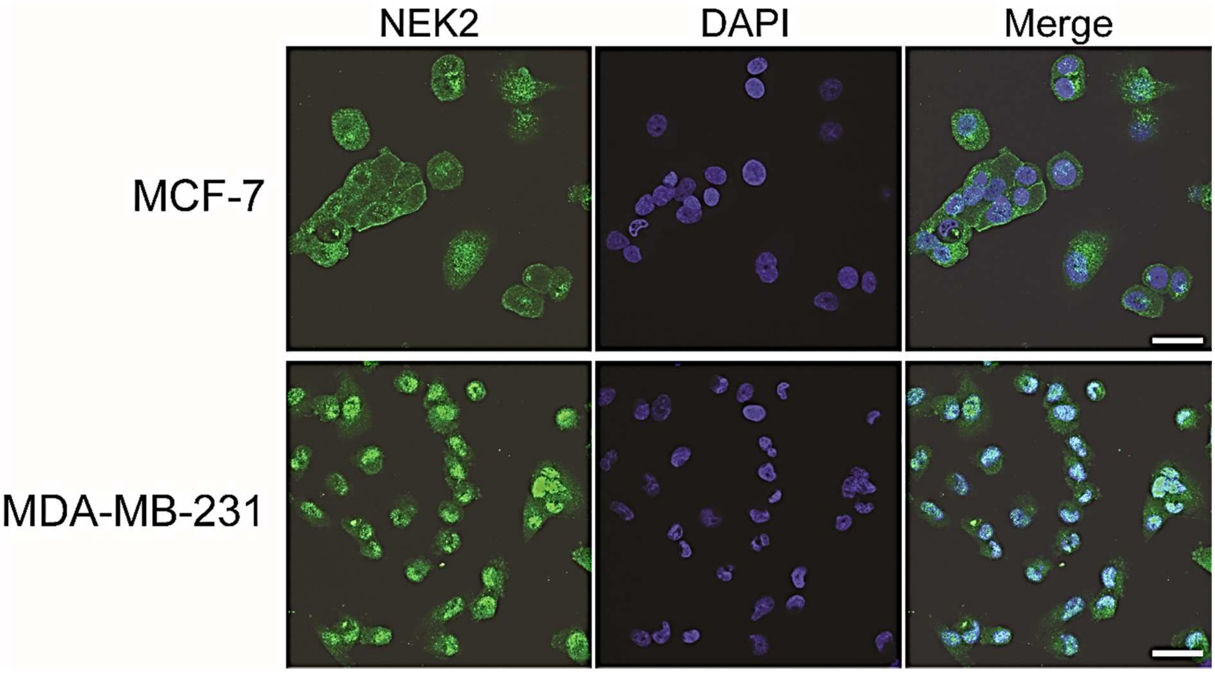

Supplemental Figure 2. NEK2 is enriched in the nucleus of triple-negative MDA-MB-231 cells. Single-channel images for confocal immunofluorescence analysis of NEK2 in MCF-7 and MDA-MB-231 cells, shown in main Figure 2. DAPI staining was used to identify nuclear morphology. Images were taken using a 60x objective lens. Scale bar 40µm.

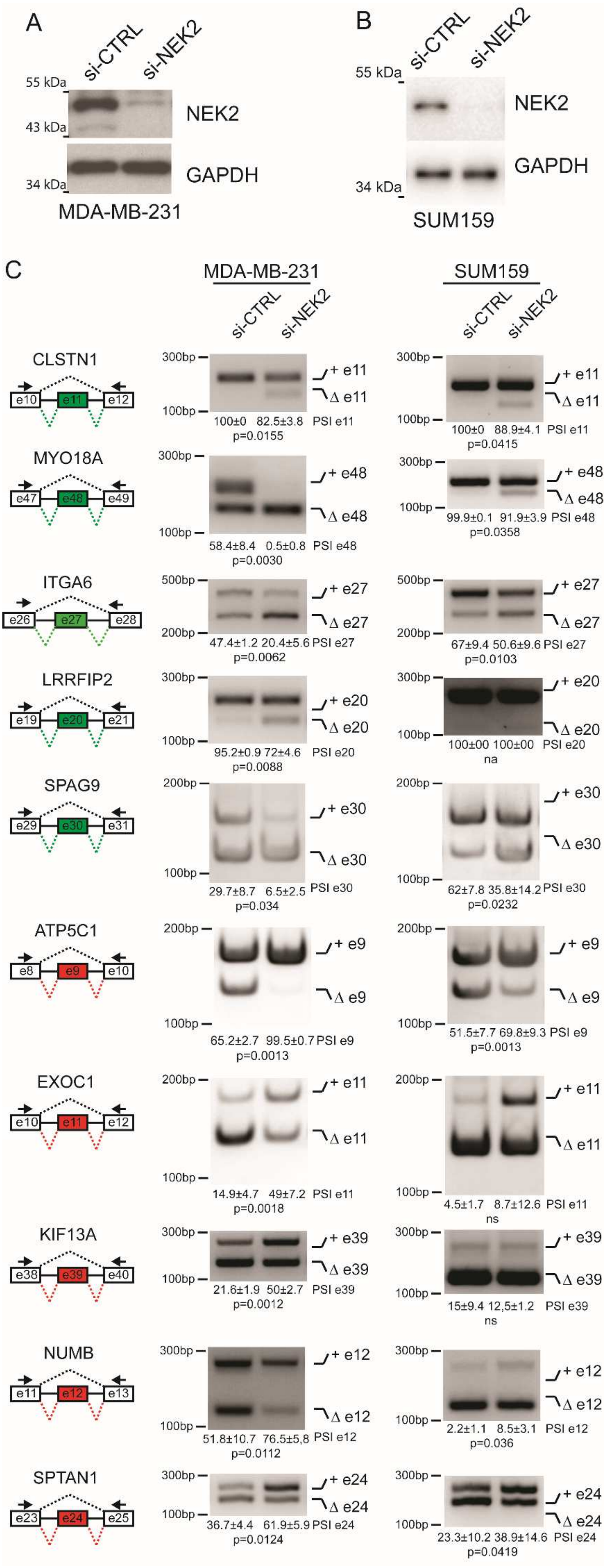
A
si-CTRL
si-NEK2
55 kDa
43 kDa
NEK2
GAPDH
34 kDa
MDA-MB-231
B
si-CTRL
si-NEK2
55 kDa
NEK2
GAPDH
34 kDa
SUM159
C
MDA-MB-231
SUM159
si-CTRL
si-NEK2
si-CTRL
si-NEK2
CLSTN1
e10 e11 e12
300bp
100bp
+ e11
Δ e11
100±0 82.5±3.8 PSI e11
p=0.0155
100±0 88.9±4.1 PSI e11
p=0.0415
MYO18A
e47 e48 e49
+ e48
Δ e48
58.4±8.4 0.5±0.8 PSI e48
p=0.0030
99.9±0.1 91.9±3.9 PSI e48
p=0.0358
ITGA6
e26 e27 e28
500bp
200bp
+ e27
Δ e27
47.4±1.2 20.4±5.6 PSI e27
p=0.0062
67±9.4 50.6±9.6 PSI e27
p=0.0103
LRRFIP2
e19 e20 e21
+ e20
Δ e20
95.2±0.9 72±4.6 PSI e20
p=0.0088
100±00 100±00 PSI e20
na
SPAG9
e29 e30 e31
200bp
100bp
+ e30
Δ e30
29.7±8.7 6.5±2.5 PSI e30
p=0.034
62±7.8 35.8±14.2 PSI e30
p=0.0232
ATP5C1
e8 e9 e10
+ e9
Δ e9
65.2±2.7 99.5±0.7 PSI e9
p=0.0013
51.5±7.7 69.8±9.3 PSI e9
p=0.0013
EXOC1
e10 e11 e12
+ e11
Δ e11
14.9±4.7 49±7.2 PSI e11
p=0.0018
4.5±1.7 8.7±12.6 PSI e11
ns
KIF13A
e38 e39 e40
+ e39
Δ e39
21.6±1.9 50±2.7 PSI e39
p=0.0012
15±9.4 12,5±1.2 PSI e39
ns
NUMB
e11 e12 e13
+ e12
Δ e12
51.8±10.7 76.5±5,8 PSI e12
p=0.0112
2.2±1.1 8.5±3.1 PSI e12
p=0.036
SPTAN1
e23 e24 e25
+ e24
Δ e24
36.7±4.4 61.9±5.9 PSI e24
p=0.0124
23.3±10.2 38.9±14.6 PSI e24
p=0.0419

Supplemental Figure 3. NEK2 regulates TNBC alternative splicing profile. A, B) Representative Western blot analysis assessing NEK2 silencing efficiency in MDA-MB-231 (A) and SUM159 (B) cells 48h post transfection with indicated control (si-CTRL) or NEK2-targeting (si-NEK2) pool of siRNAs. GAPDH was evaluated as loading control. C) PCR analysis for indicated AS events in control (si-CTRL) or NEK2 (si-NEK2) silenced MDA-MB- 231 (left panel) and SUM159 cells (right panel). Schematic representation for each event analyzed is depicted besides relative agarose gels. Green and red boxes indicate down- and up-regulated exons in si-NEK2 vs si-CTRL cells. Percentage of splicing inclusion (PSI) of indicated exons was evaluated by densitometric analysis, and results are shown below agarose gels (mean ± SD, n = 3, t test).

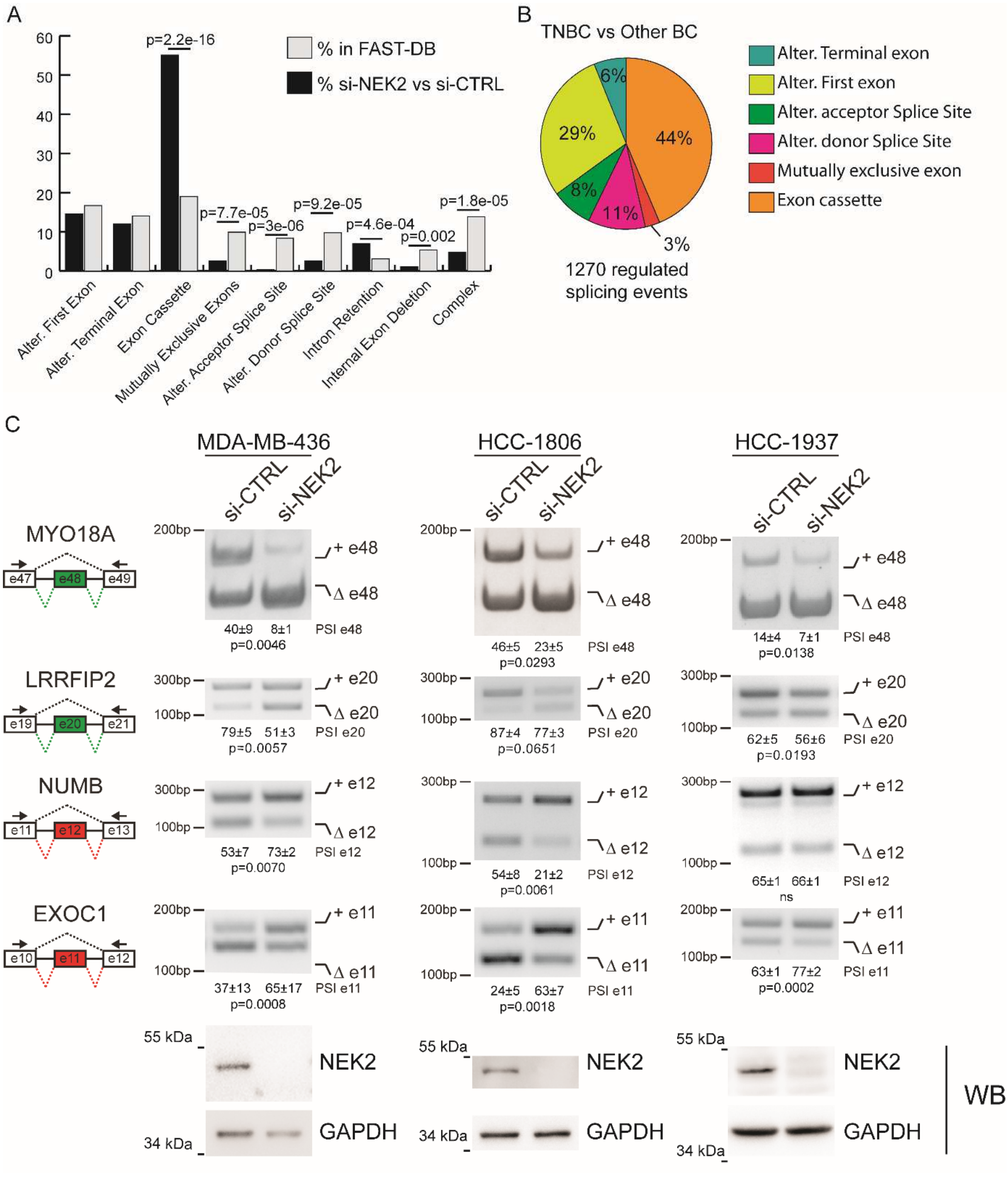

Supplemental Figure 4. NEK2 regulates TNBC specific splicing events. A) Bar graph showing comparative analysis of percentages of events annotated in FAST-DB (light gray columns) and of those differentially regulated between si-NEK2 and si-CTRL MDA-MB-231 (black columns) within each AS pattern (modified Fisher's test). B) Pie chart indicating distribution among different pattern of the regulated splicing events from the comparison of TNBC vs "Other BC" primary tumors, according to TCGA data analysis using the visual interface of the psichomics R package (|Δ median

PSI| ≥ 0.1 and FDR ≤ 0.01, Wilcoxon rank-sum test with Benjamini–Hochberg (FDR) adjustment). C) Representative PCR analysis for indicated AS events in control (si-CTRL) or NEK2 (si-NEK2) silenced MDA-MB-436 (left panel), HCC1806 (middle panel) and HCC1937 (right panel) cells. Schematic representation for each event analyzed is depicted besides relative agarose gels. Green and red boxes indicate down- and up-regulated exons in si-NEK2 vs si-CTRL cells. Percentage of splicing inclusion (PSI) of indicated exons was evaluated by densitometric analysis, and results are shown below agarose gels (mean ± SD, n = 3, t test). Representative Western blot analysis assessing NEK2 silencing efficiency 48h post transfection with indicated control (si-CTRL) or NEK2-targeting (si-NEK2) pool of siRNAs are shown below agarose gels for each cell line. GAPDH was evaluated as loading control.

A

si-NEK2 regulated cassette exons vs reference cassette exons		si-NEK2 regulated cassette exons vs constitutive exons	
Motif	Evalue	Motif	Evalue
CATGCAD	3,40E-17	CATGCAD	5,50E-20
GTTARTA	3,70E-04	KGTTARTA	1,90E-06
ACTAAYT	6,30E-04	RTKTTA	3,80E-05
RTGTTA	1,00E-02	ACTAATTK	8,30E-06
RTAAA	2,30E-02	TGAAA	9,70E-04

B

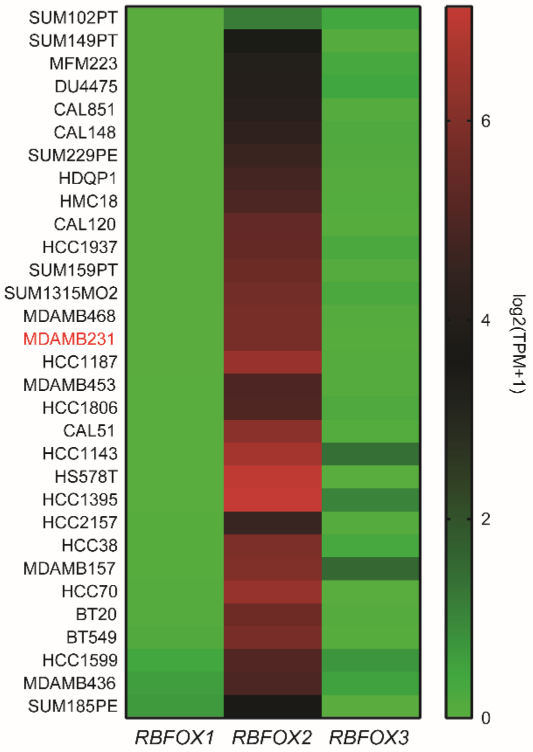

Supplemental Figure 5. CATGCAD motif for RBFOX2 is enriched within NEK2-regulated cassette exons. A) Table showing results of the search using DREME for motifs enriched within NEK2 sensitive cassette exons (si-NEK2) compared to both reference cassette and constitutive exons. E-value retrieve by DREME are indicated. B) HeatMap showing expression levels of RBFOX1, 2, 3 in a panel of $ER^-/HER2^-$ BC cell lines according to RNA-seq data from the Cancer Cell Line Encyclopedia (CCLE) project. Reference model of our study, MDA-MB-231 cell, is highlighted in red.

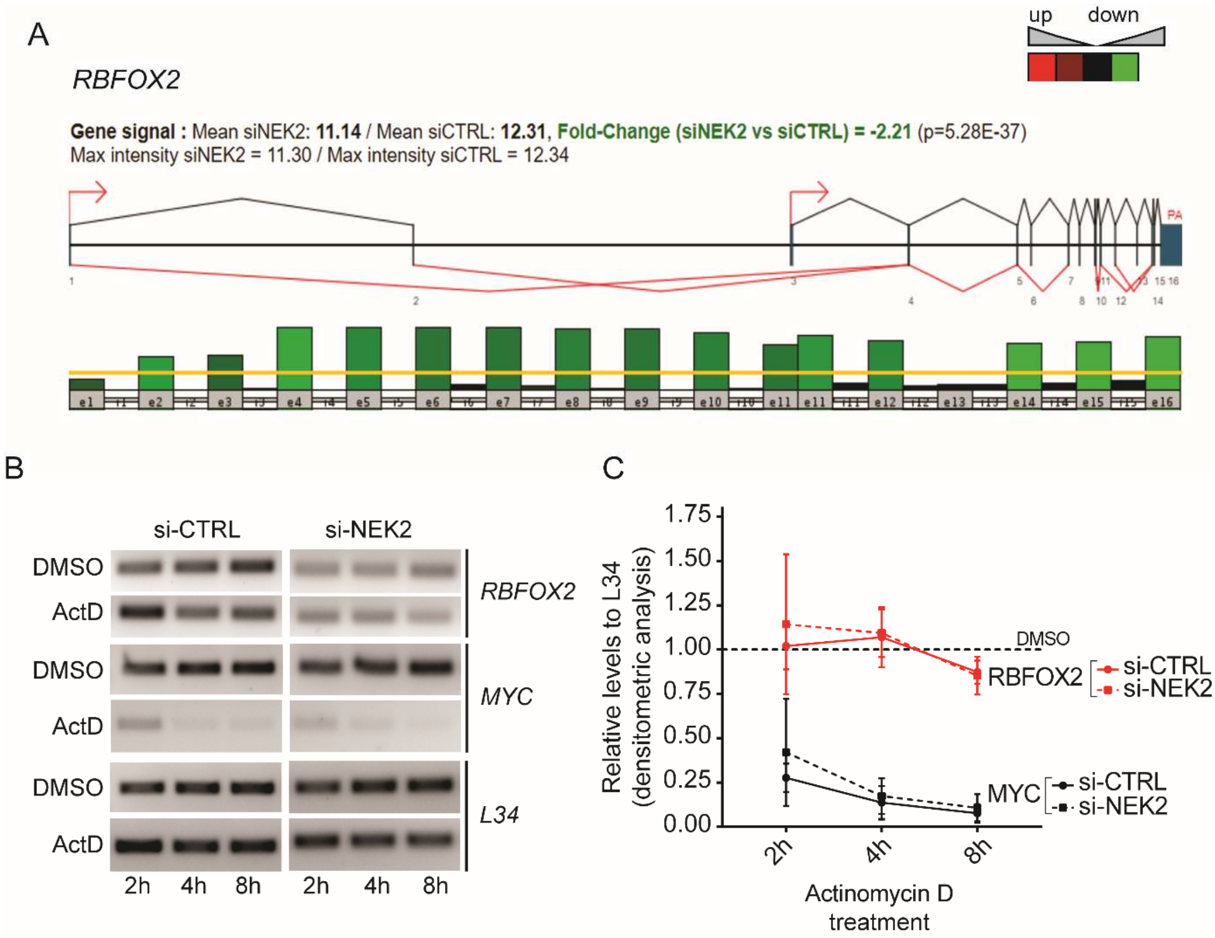

Supplemental Figure 6. NEK2 regulates RBFOX2 transcription. A) Visualization of expression profile of the RBFOX2 gene according to RNA-seq data of control and NEK2 silenced MDA-MB-231 cells. B) Representative agarose gels of RT-PCR analysis for *RBFOX2* and *MYC* transcript levels in MDA-MB-231 cell transfected with control (si-CTRL) or NEK2 targeting siRNAs (si-NEK2) and harvested at indicated time points after treatment with DMSO or Actinomycin D (ActD, 3µg/mL). Transcript levels of *L34* gene were evaluated as loading control. C) Line graph showing results of the relative levels of *RBFOX2* and *MYC* to *L34* in control or NEK2-silenced cells, treated with Actinomycin D compared to DMSO treated (set to 1), evaluated by densitometric analysis of RT-PCR experiments (n=3; mean ± SD, t-test analysis did not reveal any significant variation).

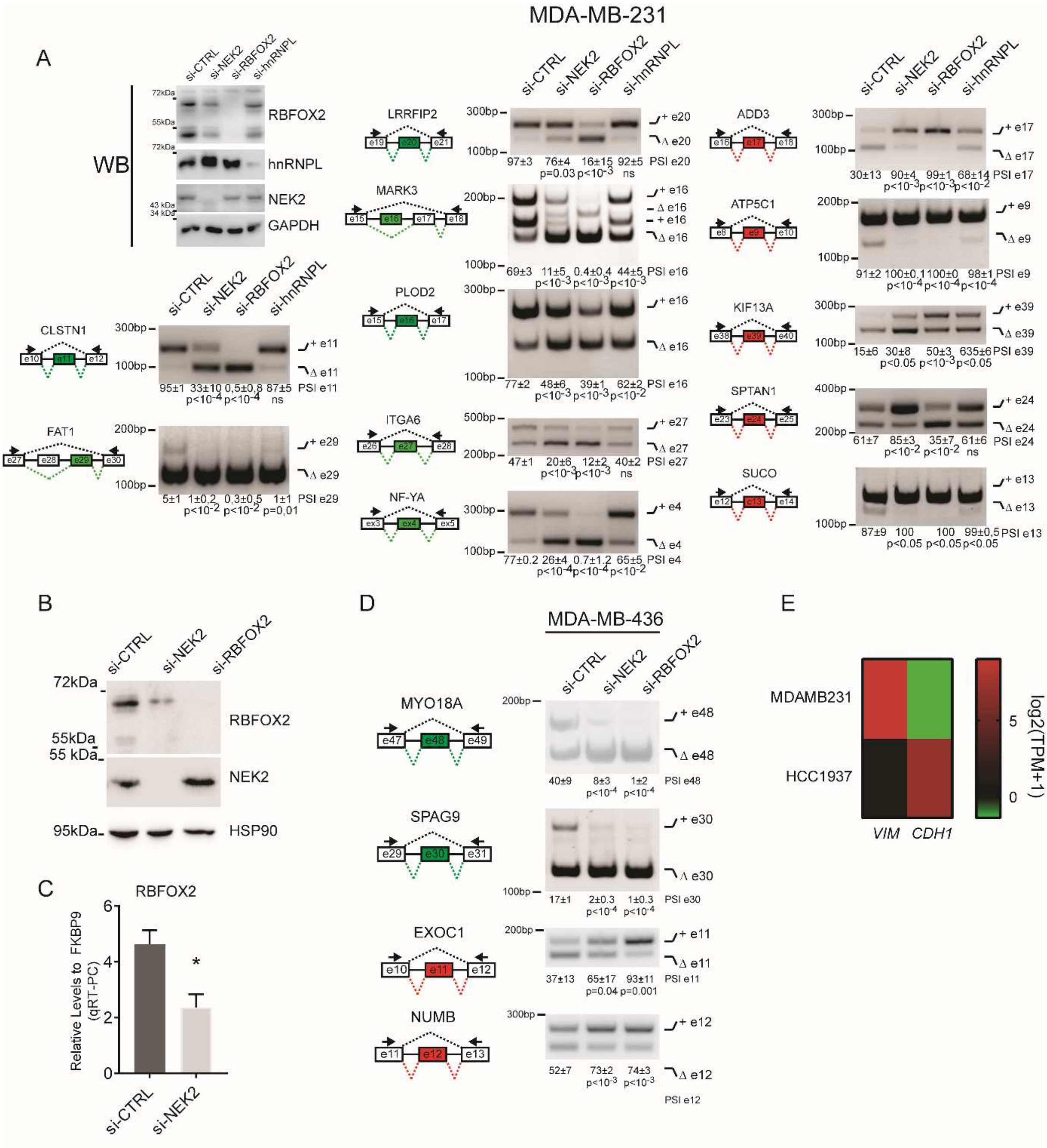

Supplemental Figure 7. NEK2 modulates an RBFOX2-dependent pro-mesenchymal splicing program in TNBC cells. A) Representative PCR analysis for indicated alternative splicing events in MDA-MB-231 cell transfected with control (si-CTRL) or NEK2, RBFOX2, hnRNPL targeting siRNAs. Schematic representation for each event analyzed is depicted beside relative agarose gels. Green and red boxes indicate down- and up-regulated exons in si-NEK2 vs si-CTRL cells. Percentage of splicing inclusion (PSI) of indicated exons was evaluated by densitometric analysis, and results are shown below agarose gels (mean ± SD, n = 3, one-way ANOVA). Upper left insert shows representative Western blot analysis assessing silencing efficiency of NEK2, RBFOX2 and hnRNPL

proteins. GAPDH was evaluated as loading control. B) Representative Western blot analysis assessing silencing efficiency of NEK2 and RBFOX2 in MDA-MB-436 transfected cells with indicated siRNAs. HSP90 was evaluated as loading control. C) Bar graph showing results of qRT-PCR analysis of the relative expression levels of RBFOX2 to FKBP9 in control (si-CTRL) or NEK2-silenced (si-NEK2) MDA-MB-436 cells (mean ± SD, n=3, *p≤0.05 t-test). D) Representative PCR analysis for indicated AS events in control (si-CTRL) or NEK2 (si-NEK2) silenced MDA-MB-436 (left panel) cells. Schematic representation for each event analyzed is depicted besides relative agarose gels. Green and red boxes indicate down- and up-regulated exons in si-NEK2 vs si-CTRL cells. Percentage of splicing inclusion (PSI) of indicated exons was evaluated by densitometric analysis, and results are shown below agarose gels (mean ± SD, n = 3, one-way ANOVA). E) HeatMap showing expression levels of the epithelial (*CDH1*) and mesenchymal (*VIM*) markers in MDA-MB-231 and HCC1939 cell lines according to RNA-seq data from the Cancer Cell Line Encyclopedia (CCLE) project.

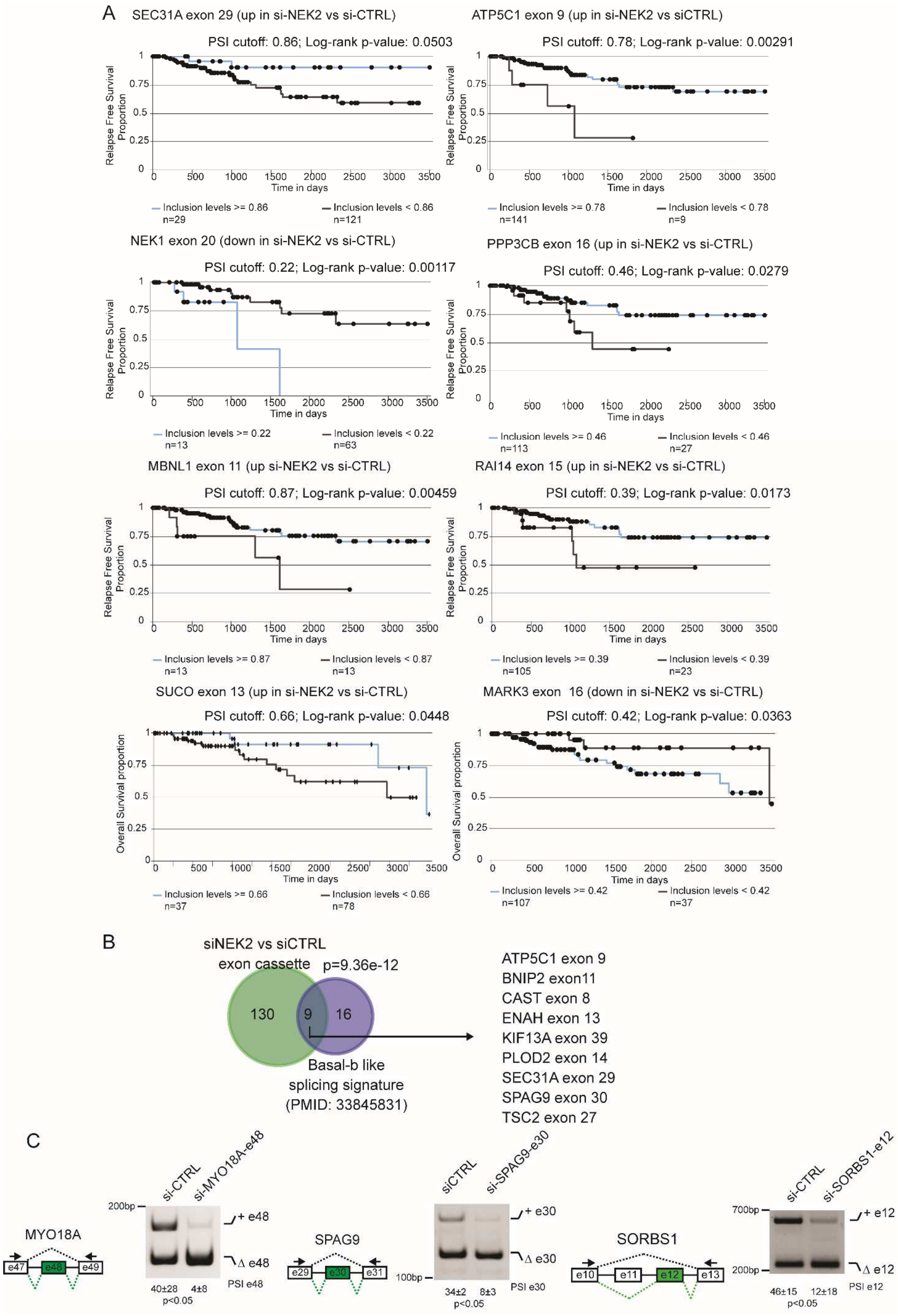
A
SEC31A exon 29 (up in si-NEK2 vs si-CTRL)
PSI cutoff: 0.86; Log-rank p-value: 0.0503
Relapse Free Survival Proportion
Time in days
Inclusion levels >= 0.86 n=29
Inclusion levels < 0.86 n=121
ATP5C1 exon 9 (up in si-NEK2 vs siCTRL)
PSI cutoff: 0.78; Log-rank p-value: 0.00291
Inclusion levels >= 0.78 n=141
Inclusion levels < 0.78 n=9
NEK1 exon 20 (down in si-NEK2 vs si-CTRL)
PSI cutoff: 0.22; Log-rank p-value: 0.00117
Inclusion levels >= 0.22 n=13
Inclusion levels < 0.22 n=63
PPP3CB exon 16 (up in si-NEK2 vs si-CTRL)
PSI cutoff: 0.46; Log-rank p-value: 0.0279
Inclusion levels >= 0.46 n=113
Inclusion levels < 0.46 n=27
MBNL1 exon 11 (up si-NEK2 vs si-CTRL)
PSI cutoff: 0.87; Log-rank p-value: 0.00459
Inclusion levels >= 0.87 n=13
Inclusion levels < 0.87 n=13
RAI14 exon 15 (up in si-NEK2 vs si-CTRL)
PSI cutoff: 0.39; Log-rank p-value: 0.0173
Inclusion levels >= 0.39 n=105
Inclusion levels < 0.39 n=23
SUCO exon 13 (up in si-NEK2 vs si-CTRL)
PSI cutoff: 0.66; Log-rank p-value: 0.0448
Overall Survival proportion
Inclusion levels >= 0.66 n=37
Inclusion levels < 0.66 n=78
MARK3 exon 16 (down in si-NEK2 vs si-CTRL)
PSI cutoff: 0.42; Log-rank p-value: 0.0363
Inclusion levels >= 0.42 n=107
Inclusion levels < 0.42 n=37
B
siNEK2 vs siCTRL exon cassette
p=9.36e-12
130
9
16
Basal-b like splicing signature (PMID: 33845831)
ATP5C1 exon 9
BNIP2 exon11
CAST exon 8
ENAH exon 13
KIF13A exon 39
PLOD2 exon 14
SEC31A exon 29
SPAG9 exon 30
TSC2 exon 27
C
MYO18A
si-CTRL
si-MYO18A-e48
200bp
+ e48
Δ e48
40±28
4±8
p<0.05
PSI e48
SPAG9
siCTRL
si-SPAG9-e30
100bp
+ e30
Δ e30
34±2
8±3
p<0.05
PSI e30
SORBS1
si-CTRL
si-SORBS1-e12
700bp
200bp
+ e12
Δ e12
46±15
12±18
p<0.05
PSI e12

Supplemental Figure 8. NEK2-induced splice variants promote a mesenchymal phenotype in TNBC cells. A) Representative Kaplan–Meier survival curves illustrating the relapse free survival or overall survival probability of TNBC patients stratified according to inclusion levels of indicated NEK2-sensitive cassette exons. PSI-cut-off and p-value are indicated above each graph. Analysis and graphs were drawn by visual interface of the psichomics R package. B) Venn diagram showing a significant overlap (hypergeometric distribution test) between NEK2-regulated cassette exons and the minimal splicing signature identifying basal-b like breast cancers from the indicated study. Genes and cassette exons in the overlap are indicated. C) PCR analysis evaluating silencing efficiency of specific splice-variants of *MYO18A*, *SPAG9* and *SORBS1* gene upon transfection of si-RNAs targeting indicated exons. Schematic representation for each event analyzed is depicted besides relative agarose gels. Percentage of splicing inclusion (PSI) of indicated exons was evaluated by densitometric analysis, and results are shown below agarose gels (mean ± SD, n = 4, t-test).
